# Protective effects of Astragalus membranaceus polysaccharide against aluminum oxide nanoparticle-induced growth retardation and oxidative-immunological disruption in *Oreochromis niloticus*

**DOI:** 10.1038/s41598-026-46411-2

**Published:** 2026-04-13

**Authors:** Ola Hasan Abd El Megeed, Maha M. Rashad, Ghada E. Ali, Eman Ragab, Ibrahim Elmaghraby, Fady Sayed Youssef, Sahr B. Mahmoud

**Affiliations:** 1 Department of Aquatic Animal Medicine and management, Faculty of Veterinary Medicine, Cairo University, Giza, 12211 Egypt; 2 Department of Biochemistry and Molecular Biology, Faculty of Veterinary Medicine, Cairo University, Giza, 12211 Egypt; 3 Department of Microbiology, Faculty of Veterinary Medicine, Cairo University, Giza, 12211 Egypt; 4 Department of Pathology, Faculty of Veterinary Medicine, Benha University, Toukh, 13736 Egypt; 5 Department of Pharmacology, Faculty of Veterinary Medicine, Cairo University, Giza, 12211 Egypt; 6 Department of Hydrobiology, Veterinary Research Institute, National Research Centre, Dokki, Giza, Egypt

**Keywords:** Aluminum oxide nanoparticles- induced toxicity, Astragalus polysaccharides, Oxidative stress, Immune genes, *Oreochromis niloticus*, Biochemistry, Physiology, Zoology

## Abstract

Although the toxicity of aluminum-based nanoparticles (Al_2_O_3_-NPs) in fish has been widely studied, strategies to mitigate their harmful effects remain limited. Moreover, the modulatory role of Astragalus membranaceus polysaccharides (APS) under this toxic stress is still poorly understood, despite the widespread presence of Al_2_O_3_-NPs in aquatic ecosystems and the need for effective mitigation strategies for sustainable aquaculture. In this study*, Oreochromis niloticus* was exposed to Al_2_O_3_-NPs (10 mg/L) with or without dietary APS (1.5 or 3 mg/kg) for four weeks. A total of 270 fish (initial weight = 25.28 ± 0.086 g) were randomly distributed into six triplicate groups (*n* = 15 per group) as control, APS (1.5 mg/kg–3 mg/kg), Al_2_O_3_-NPs, and two co-treated groups (Al_2_O_3_-NPs + APS). Al_2_O_3_-NPs exposure showed significant effects on growth performance compared to the control group (*P* < 0.05), as shown by the decrease in final weight (FW: -9.4%), weight gain (WG: − 21.4%), specific growth rate (SGR: -17.1%), along with an increase in feed conversion ratio (FCR: + 16.9%). Liver function was severely affected, as evidenced by an increase in ALT levels (+ 39%) and decrease in total protein (− 26.5%) and IgM levels (− 19.3%) (*P* < 0.05). In addition, the expression of antioxidant-related genes, such as *CAT* and *SOD*, was downregulated, while the expression of pro-inflammatory cytokines, such as *TNF-β* and *IL-1β*, as well as stress-related gene, such as *MT*, was upregulated in the liver and gills (*P* < 0.05). Histopathological examination showed noticeable degenerative and necrotic changes in the liver, kidney, muscle, gill, and spleen tissues. Co-treatment with APS, particularly at 3 mg/kg, significantly improved growth parameters (FW, 20.3% increase; WG, 53.1% increase; and SGR, 38.8% increase) (*P* < 0.05), ameliorated hepatic and renal histopathological changes, restored antioxidant gene expression and normalized inflammatory mediators compared with the Al_2_O_3_-NPs-exposed group. Compared with the Al_2_O_3_-NPs–exposed group, the survival rate increased by 5.3%, ALT levels decreased by 60.1%, while total protein and IgM levels increased by 29.6% and 8.9%, respectively. Overall, APS supplementation effectively mitigated Al_2_O_3_-NPs-induced toxicity in *O. niloticus* by regulating antioxidant capacity, suppressing inflammatory responses, and improving tissue structure, highlighting its potential as an eco-friendly functional food additive to protect fish health under nano-toxic stress*.*

## Introduction

Over the past decades, aquaculture has become a major source of affordable, high-quality protein for human consumption^1,2^. However, this sector faces increasing challenges from environmental pollution, leading to stress conditions and recurrent disease outbreaks^3,4^. Marine and freshwater environments are widely contaminated with heavy metals, pesticides, and various other pollutants, which adversely affect the growth and health of aquatic animals^5,6^. Among these contaminants, Aluminum nanoparticles (Al_2_O_3_-NPs) are increasingly present in aquatic bodies due to their use in nutrient and vaccine delivery, water purification, and fish breeding^7,8^.

*Oreochromis niloticus* was selected as a model organism due to its fast growth, ease of cultivation, and economic importance in aquaculture. Moreover, it is sensitive to environmental pollutants, making it a suitable species to study nanoparticle-induced toxicity^9^. Exposure to Al_2_O_3_-NPs has been associated with oxidative stress, immune dysfunction, behavioral abnormalities, neurotoxicity, and impaired growth in *Oreochromis niloticus*^10–21^.

Key organs such as gills, liver, kidney, spleen, and muscles are more susceptible to the toxic effects of Al_2_O_3_-NPs because they are involved in the processes of uptake, detoxification, excretion, and immune response, and also in the potential bioaccumulation of NPs. These organs are the targets for the assessment of the toxicity of nanoparticles. Gills, with their large surface area and thin epithelium, are the main entry point for nanoparticles and sensitive indicators of early toxic damage due to ROS generation^6^. The liver, as the central detoxification organ, is vulnerable to oxidative stress and inflammation. The kidney, responsible for excretion and osmoregulation, may accumulate nanoparticles leading to tissue damage. The spleen, a major lymphoid organ, reflects systemic immune responses, while muscle tissue indicates overall distribution and potential bioaccumulation.

Recently, medicinal plants and their extracts have gained attention as natural feed additives to enhance growth, immune responses, and stress alleviation in aquaculture^22–25^. *Astragalus membranaceus*, a Chinese medicinal herb, rich in saponins, flavonoids, and polysaccharides has been reported to enhance immune responses, antioxidant defense, metabolism, and overall health of fish^25–27^. APS exhibits antioxidant activity, mitigating reactive oxygen species (ROS) and protecting against oxidative stress in *Micropterus salmoides* and carp erythrocytes^28,29^. Additionally, APS has been shown to enhance immune responses, growth performance, and digestive efficiency in fish species such as *Micropterus salmoides*, *Lateolabrax maculatus*, *Oreochromis niloticus*, *Channa argus*, and *Cyprinus carpio*^30–35^.

Despite the reported antioxidant and immunomodulatory activities of Astragalus polysaccharides (APS), their protective effects against aluminum oxide nanoparticles (Al_2_O_3_-NPs)-induced toxicity have not been fully investigated. In particular, there is a lack of integrated studies addressing oxidative stress, inflammation, and histopathological alterations in a single experimental design.

Thus, the present study was designed to assess the potential of dietary APS in mitigating Al_2_O_3_-NPs-induced toxicity in *O. niloticus* with particular attention to antioxidant enzymes (CAT and SOD), pro-inflammatory cytokines (TNF-β and IL-1β), and stress proteins (MT). This study provides mechanistic insights into the use of APS as a functional feed additive and highlights its potential application in improving fish health and sustainability in aquaculture systems.

## Materials and methods

### Ethics approval and consent to participate

The study protocol and all experimental procedures were permitted by the Research Ethics Committee of the Faculty of Veterinary Medicine, Cairo University, Egypt (Vet CU301220251273). All methods were performed in accordance with the relevant guidelines, regulations, and ARRIVE standards.

### Preparation and preliminary phytochemical testing of *Astragalus membranaceus* root extract (AMRE)

High-purity *Astragalus membranaceus* root powder (Qi Jing Ltd., Beijing, China) was processed and extracted at the Department of Pharmacology, Faculty of Veterinary Medicine, Cairo University. Ethanol (CAS 64-17-5), calcium oxide (CaO) (CAS 1305-78-8), and sodium carbonate (Na_2_CO_3_) (CAS 497–19-8) were purchased from Sigma-Aldrich (USA). The aqueous root extract of *A. membranaceus* was prepared via evaporation, following the protocol of^37^. Briefly, 100 g of the root powder was reflux-extracted with distilled water (solid–liquid ratio, 1:10, w/v) three times for 2 h each. The combined aqueous extracts were filtered and evaporated to the desired volume, after which four volumes of absolute ethanol were added to precipitate the polysaccharides. The precipitate was incubated at 4 °C overnight and then centrifuged at 4000 rpm for 20 min. The supernatant was dried under reduced pressure to obtain the crude aqueous extract of *A. membranaceus* root. Phytochemical constituents in the crude extract were identified using the standard protocols described by Pant et al.^38^.

### Calculating the amount of total phenolic and flavonoids in *Astragalus membranaceus* root

Total phenolic content (TPC) of the *Astragalus membranaceus* (AM) extract was determined spectrophotometrically using the Folin–Ciocalteu method according to the procedure described by Baba and Malik^39^. The AM extract was diluted to 1 mg/mL in distilled water (3 mL) followed by the addition of 0.5 mL of Folin–Ciocalteu reagent. The mixture was mixed well for 3 min, and then 2 mL of 20% (w/v) sodium carbonate solution was added. The resulting reaction mixture was further incubated in the dark for 60 min. Absorption was measured at 650 nm. TPC was estimated from a calibration curve of gallic acid and expressed as micrograms of gallic acid equivalents (µg GAE/g dry AM) using a regression equation y = 105.51× + 2.1824 (R^2^ = 0.9978).

TFC was evaluated by the aluminum chloride colorimetric assay according to the method of Baba and Malik^39^. The reaction mixture containing 1 mL of AM extract, 3 mL of methanol, 0.2 mL of 10% aluminum chloride, 0.2 mL of 1 M potassium acetate, and 5.6 mL of distilled water was incubated at room temperature for 30 min. The absorbances were measured at 420 nm. Rutin (1 mg/mL) was used as reference standard, and flavonoid content was calculated from the standard calibration curve (y = 0.0031x + 0.0188; R^2^ = 0.9959) and expressed as gram of rutin equivalents per gram of dry AM (g RE/g dry AM).

### Preparation of aluminum oxide nanoparticles (Al_2_O_3_-NPs)

Aluminum oxide (Al_2_O_3_) nanoparticles (Al_2_O_3_-NPs; ≥ 99% purity; particle size 20–50 nm) were purchased from Sigma-Aldrich (St. Louis, MO, USA) through a local distributor in Egypt. They were dispersed in distilled water and sonicated for 30 min prior to experimental use to ensure proper suspension and homogeneity. Other reagents and solvents of analytical grade were obtained from standard commercial suppliers.

### Characterization of nanoparticles

The synthesized nanoparticles were characterized for particle size, zeta potential, and morphology. Particle size was determined by Dynamic light scattering (DLS) while zeta potential was analyzed using a Zetasizer Nano ZS (Malvern, UK). The morphology was examined by TEM (JEOL JEM-2100) according to Sharma et al.^40^ and Yallapu et al.^41^.

### Fish maintenance and experimental outlines

This experiment was carried out at the Hydrobiology Department, National Research Centre, Dokki, Giza. A total of 270 healthy juvenile Nile tilapia, with an average initial body weight of 25.28 ± 0.08 g, were purchased from a private fish farm in Fayoum Governorate, Egypt. Upon arrival, fish were transferred to the laboratory, placed in rectangular glass tanks (40cm*25cm*70cm, 100L capacity filling point 70L), and acclimated for two weeks while being fed a standard basal diet. At the time of acclimation, water parameters were maintained at a temperature of 29 ± 1.09°C, dissolved oxygen of 7 ± 0.60 mg/L, pH of 7.3 ± 0.6, total ammonia nitrogen of 0.03 ± 0.02 mg/L, and nitrite of 0.03 ± 0.01 mg/L with a photoperiod of 12:12 h light/dark. Subsequently acclimation, fish were randomly distributed into tanks (*n* = 15 fish / group, three replicates per treatment) under continuous water flow. Fish were fed three times a day (3% of their body weight) for 30 days^42^. The extruded diets were purchased from Aller Aqua Feed (Aller® Aqua, 6^th^ of October City, Egypt) and prepared according to NRC^43^ recommendation. As shown in Table 1, the chemical composition of the diet was around: crude protein (30%), crude fat (6%), crude fiber (5%), nitrogen-free extract (41%), ash (8%), and gross energy (3,100 kcal/kg). The control group was supplemented with the basal diet only. Second and third groups were supplemented with 1.5g/kg and 3g APS /kg diets, respectively. Fourth group exposed to 10 mg/L Al_2_O_3_-NPs in water along with the basal diet. Fifth and sixth groups were co-treated in the same manner with 10 mg/L Al_2_O_3_-NPs and were supplemented with 1.5g and 3g APS /kg diets, respectively. The Al_2_O_3_-NPs exposure dose of 10 mg/L was based on previous studies indicating sub-lethal toxicity to *O. niloticus*^10^, and the levels of APS added to the diet (1.5 and 3 g/kg) were based on an effective growth-promoting and antioxidant capacity in fish, as demonstrated by Lin et al.^44^. Throughout the 4-week experiment, fish were observed for reflex responses, behavioral changes, any abnormalities as well as daily mortalities were recorded. Uncleaned water was partially replaced (30% of tank water) with dechlorinated water and Al_2_O_3_-NPs replenished twice a week to maintain uniform concentrations.

**Table 1:** Fish basal diet (raw ingredients, chemical composition, and bioactive compounds).

Category	Component	Inclusion level (%)
Raw ingredients (100%)	Soybean meal	30.0
	Fish meal	13.0
	Yellow corn	28.0
	Wheat bran	15.0
	Rice bran	8.0
	Vegetable oil (soybean oil)	2.0
	Dicalcium phosphate (DCP)	2.0
	Salt (NaCl)	0.5
	Vitamin-mineral premix	1.0
	Methionine + Lysine	0.5
	Total	100.0
Chemical composition	Crude protein	30.0
	Crude lipid	6.0
	Crude fiber	5.0
	Ash	8.0
	Moisture	10.0
	Nitrogen-free extract (NFE)	41.0
	Calculated gross energy	3100 kcal/kg
Bioactive compounds	APS g/kg diets	1.5 and 3
	Total phenolic (µg GAE/g dry extract ± SD)	36.2 ± 1.1
	Total flavonoid (mg RE/g dry extract ± SD)	81.3 ± 2.7

*Vitamin and mineral content (per kg of mixed product): cobalt, 30 mg; folic acid, 220 mg; vitamin B1, 1300 mg; pantothenic acid, 4000 mg; antioxidant, 0.63 g; biotin, 140 mg; selenium, 65 mg; iron, 835 mg; zinc, 17,300 mg; iodine, 110 mg; niacin, 5000 mg; vitamin B6, 2465 mg.

### Assessment of growth indices

At the beginning of the experimental study, the initial body weight (g) of all fish was determined. Every week fish groups were weighed to adjust the diet and to check the growth parameters. Upon completion of the experimental period, fish were weighed then weight gain percent and specific growth rate (SGR, %/d) were calculated. Feed intake and feed conversion ratio (FCR) were measured according to Abdel-moneam et al.^45^ and Li et al.^46^, as follows:

- **Weight gain (g)** = final body weight (FBW) (g)—initial body weight (IBW) (g)
- **Weight gain rate %** = [final body weight (FBW) (g)—initial body weight (IBW) (g)/initial body weight] × 100,
- **Specific growth rate (%/d)** = 100 × [Ln final body weight (g)—Ln initial body weight (g) / number of feeding days],
- **Feed conversion ratio** = Feed intake (g)/Body weight gain (g),
- **Fish survivability** was calculated as follows, Survival rate% = (number of fish counted/number of stocked fish) × 100.

### Water quality parameters

Throughout the experimental trial, water quality parameters were monitored weekly. Dissolved oxygen (DO) was measured using an OXY-CHECK probe, whereas total ammonia nitrogen (TAN), nitrite (NO_2_), and nitrate (NO_3_) contents were analyzed using a colorimetric kit. PH and electrical conductivity (EC) of the water were measured by using a digital water tester (C-600, HANNA Instruments).

### Blood sampling, serum separation, and organ collection

Upon termination of the experimental period, all fish groups were fasted for 24 h before blood collection. Then, fish were anaesthetized using clove oil (50 μL/L; Algomhuria Co., Egypt)^47^. Deep anesthesia was confirmed by loss of reflexes before sampling. Following deep anesthesia, blood samples were collected from the caudal vein of 5 fish / replicate on non-heparinized tubes. Tubes were left standing upright on ice for 4 h to allow clotting. The clotted samples were then centrifuged at 3,500 rpm for 15 min at 4 °C to obtain the serum. The serum was harvested and stored at -20 °C until biochemical analysis. Following blood collection, fish were humanely euthanized by prolonged immersion in an overdose of clove oil. Liver, kidney, gills, spleen, and muscle tissues were carefully excised for subsequent biochemical, histopathological, and molecular analyses.

### Serum immunoglobulin M (IgM)

Following the experimental period, serum immunoglobulin M (IgM) concentrations were measured in 5 fish /replicate according to the manufacturer’s instructions of the ELISA kit (Biocheck Inc., USA). The optical density of the samples was recorded at 490 nm with a SpectraMax 190 spectrophotometer (Molecular Devices, USA). Sample IgM concentrations, in mg/dL, were derived by comparing their optical density readings with the control.

### Biochemical tests

Following the methods of Reinhold et al.^48^, total protein (TP) and albumin (ALB) levels were measured using commercial kits (Spectrum Co., Egypt) (Catalogue numbers: 310001 and 21100, respectively). Globulin (GLO) concentration was calculated by subtraction of ALB from TP. Alanine aminotransferase (ALT) activity was measured according to the manufacturer’s procedure (Bio Diagnostic Co., Egypt) using a Unico S-1000 spectrophotometer.

### Quantitative real-time PCR analysis

mRNA expression values of antioxidant-related genes (*catalase, CAT; superoxide dismutase, SOD*), immune-related mediators (*tumor necrosis factor-beta, TNF-β; interleukin-1 beta, IL-1β*), as well as the gene metallothionein (*MT*) in both gill and liver samples were analyzed by quantitative real-time PCR (qRT-PCR). Gene expression values were assessed by analyzing cDNA samples, in which *glyceraldehyde-3-phosphate dehydrogenase*, *GAPDH* served as the reference gene, as mentioned by Abdel-moneam et al.^45^. Briefly, total RNA was extracted using an extraction kit (Applied Biotechnology, EX02), and the amount and purity of the RNA were evaluated using a NanoDrop spectrophotometer. Complementary DNA was then generated using the extracted RNA through reverse transcription (Applied Biotechnology, AMP 11). Quantitative PCR amplification was then carried out using the SYBR Green PCR Master Mix Kit (Applied Biotechnology, AMP 03). Gene expression analysis was done using target gene expression normalized to *GAPDH* expression Abdel-Moneam et al.^49^. Fold expression was also evaluated using the comparative 2^−ΔΔ^Ct method of Livak and Schmittgen^50^. Primer sequences utilized in the analysis are also indicated in Table 2.

**Table 2:** The primer sequences used in RT-PCR analysis.

Gene	Accession number	Forward	Tm	Reverse	Tm
*GAPDH*	JN381952.1 Abd El Megeed et al.^51^	GCTGTACATGCACTCCAAGG	58.91	ACTCAAACACACTGCTGCTG	58.98
*CAT*	JF801726.1 Abdel-moneam et al.^45^	AGAACTTGGCCGGGTTTCTA	58.94	CGGCTGTAAACGTGCAAAGT	59.69
*SOD*	JF801727.1 Abdel-moneam et al.^49^	CCCTACGTCAGTGCAGAGAT	58.89	GCCGCCTCCATTAAACTTGA	58.54
*TNF- β*	NM_001279533.1 Abd El Megeed et al.^51^	GCCTCACAATTCTCAGCCAC	59.19	AAACACGCCAAAGAAGGTCC	58.97
*IL-1β*	KF747686.1 Abd El Megeed et al.^48^	CACAAGGATGACGACAAGCC	59.20	TCTCCTGACACACTTCCACC	58.95
*MT*	XM_003447045.5	CCGAAGAGACAAGAGCAACG	58.94	CTGGTGTCGCATGTCTTTCC	59.20

Primer sets designed by free online software Primer3 (v. 0.4.1.0) http://bioinfo.ut.ee/primer3-0.4.0. *Glyceraldehyde-3-phosphate dehydrogenase (GAPDH), Catalase (CAT), Superoxide Dismutase (SOD), Tumor necrosis factor-α (TNF- β), Interleukin-1β (IL-1β), metallothionein (MT).*

### Histopathological examination

The target organs (gills, muscle, liver, kidney, spleen) of Nile tilapia were carefully dissected and histologically prepared. Tissues were fixed in 10% neutral buffered formalin for 48 h at room temperature, then dehydrated in graded ethanol of increasing concentration (50%, 70%, 90%, and 100%) and paraffin-embedded at 60 °C. Sections of 5 μm thickness were prepared by using a microtome (YD-355AT Fully Automatic Microtome, China). Tissue sections were stained with hematoxylin and eosin (H&E) to observe general morphological features^52^. All sections were inspected for pathological alterations using a Nikon E800 Eclipse light microscope, and representative photomicrographs were captured using an integrated digital camera. Histopathological assessment was conducted on H&E-stained sections of gill, muscle, and kidney tissues. Ten distinct microscopic fields per specimen were analyzed for each organ using light microscopy at standardized magnifications (× 200). A semiquantitative scoring system from 0 to 3 was used to rate the severity of the lesions. A score of 0 meant normal histology, 1 meant mild focal changes, 2 meant moderate multifocal changes, and 3 meant severe diffuse changes^53^. The following lesion categories were assessed:

Gill: lamellar hyperplasia, lamellar fusion, epithelial lifting, and vascular congestion.

Muscle: myocyte degeneration, Zenker’s necrosis, interstitial edema, and muscular atrophy.

Kidney: glomerular atrophy, tubular degeneration, vascular congestion, and epithelial desquamation.

Scores for each lesion type were recorded across ten fields, and composite totals were calculated per organ system. All evaluations were performed blindly by two independent observers to minimize bias.

### Morphometric analysis

A quantitative morphometric analysis was conducted to assess hepatocyte vacuolization in the liver and melanomacrophage centers in the spleen. ImageJ software (NIH, USA) was used to look at ten representative fields on each slide. We used the “Color Threshold” tool to separate vacuolar spaces from hepatic parenchyma and the “Area Fraction” function to find out how much of the field was taken up by vacuoles. To find the proportion of the field that was taken up by melanomacrophage centers, pigmented areas were separated using thresholding, and the area fraction (%) was calculated.

### Statistical analysis

Data were statistically analyzed using SPSS version 20. One-way ANOVA established experimental group differences, and post hoc multiple comparisons were conducted using Tukey’s honest significant difference (HSD) test. Results are expressed as mean ± standard error of the mean (SEM) of five replicates (*n* = 5).

## Results

### Phytochemical screening of *Astragalus membranaceus* root extract (AMRE)

**Fig. 1:**
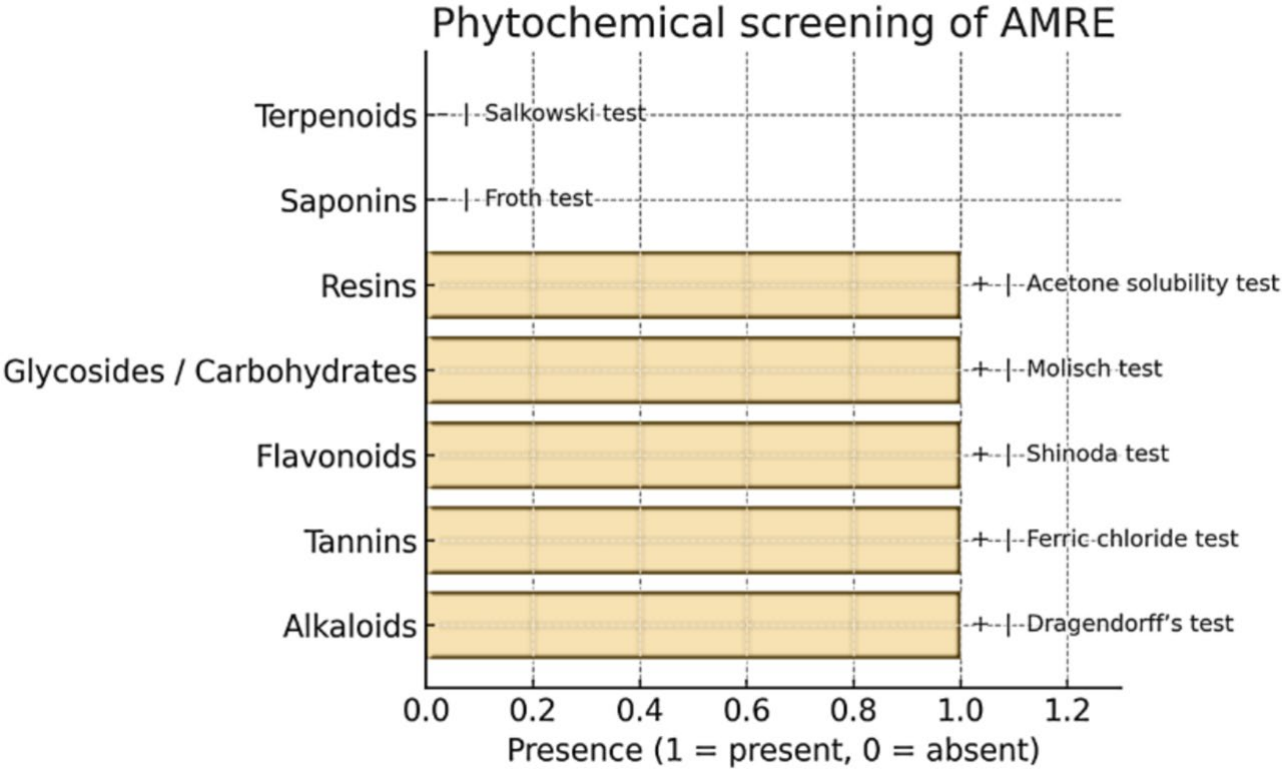
Phytochemical test detection in AMRE: “ + ” present and “–” absent; tests employed were Dragendorff’s (alkaloids), ferric chloride (tannins), Shinoda (flavonoids), Molisch (glycosides), acetone solubility (resins), froth, Salkowski finger/thin layer plate/TLC (depending on the availability) for terpenoids.

As shown in Table 3 and Fig. 1 preliminary phytochemical screening revealed the presence of major secondary metabolites such as alkaloids, tannins, flavonoids, carbohydrates, glycosides and resins while saponins and terpenoids were absent. These findings suggest that the aqueous-ethanolic AMRE extract has a broad phytochemical composition typical of the traditional pharmacological profile of *Astragalus membranaceus.*

**Table 3:** Phytochemical screening of AMRE indicated the presence of various bioactive groups, which might be responsible for its antioxidant and antimicrobial activities.

Phytochemical constituent	Test performed	Presence (+)/absence (−)
Alkaloids	Dragendorff’s test	+
Tannins	Ferric chloride test	+
Flavonoids	Shinoda test	+
Glycosides/carbohydrates	Molisch test	+
Resins	Acetone solubility test	+
Saponins	Froth test	−
Terpenoids	Salkowski test	−

### Total phenolic content (TPC) and total flavonoid content (TFC)

As shown in Table 4 the total phenolic content of AMRE was calculated based on gallic acid standard curve (y = 105.51x + 2.1824, R^2^ = 0.9978). The average absorbance value of the extract at 650 nm was recorded to be 0.384 ± 0.012, corresponding to the TPC as 36.2 ± 1.1 µg GAE/g dry extract. The total flavonoid content was determined using rutin as a standard via the AlCl_3_ colorimetric assay (y = 0.0031x + 0.0188, R^2^ = 0.9959). The average absorbance of AMRE was 0.271 ± 0.009 at 420 nm, with TFC of 81.3 ± 2.7 mg RE/g dry extract.

**Table 4:** The total phenolic content (TPC) and total flavonoid content (TFC) of AMRE.

Total phenolic content (TPC)				
Sample	Absorbance (650 nm)	Regression equation	R^2^	TPC (µg GAE/g dry extract ± SD)
AMRE	0.384 ± 0.012	y = 105.51x + 2.1824	0.9978	36.2 ± 1.1
Total flavonoid content (TFC)				
Sample	Absorbance (420 nm)	Regression equation	R^2^	TFC (mg RE/g dry extract ± SD)
AMRE	0.271 ± 0.009	y = 0.0031x + 0.0188	0.9959	81.3 ± 2.7

### Comparisons between TPC and TFC

**Fig. 2:**
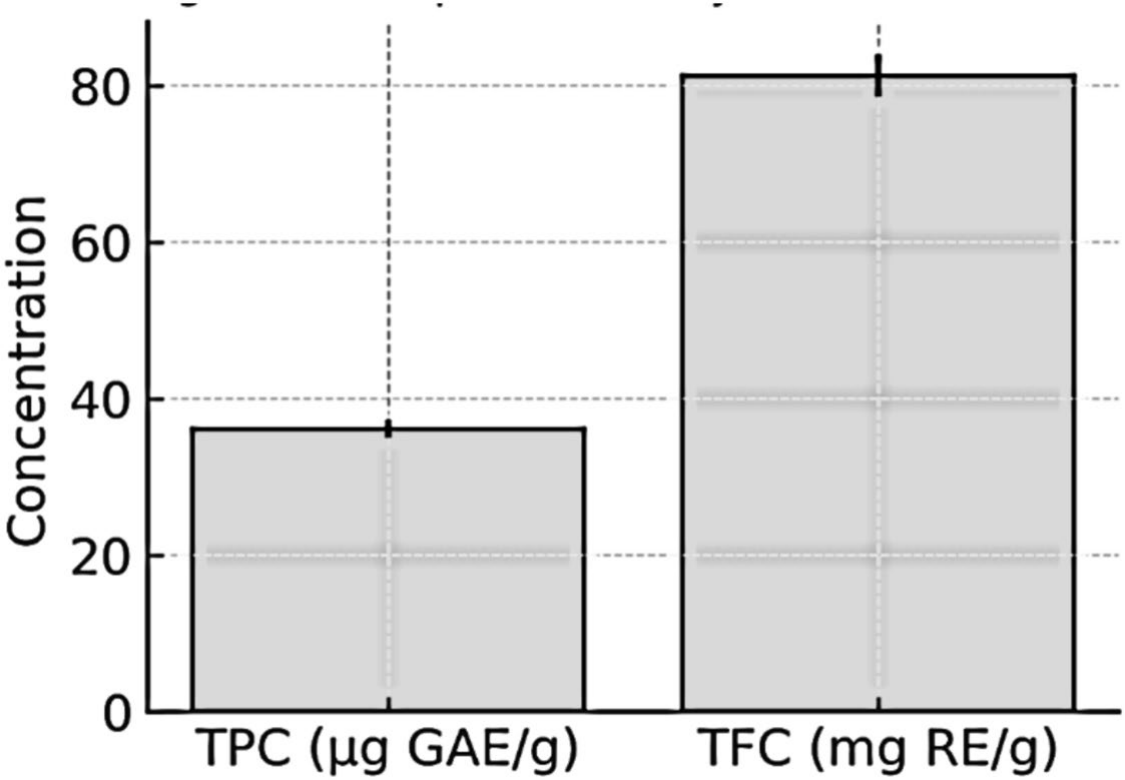
AMRE extract Total phenolic (TPC) and flavonoid (TFC) contents Comparative bar chart.

The highest polyphenolic fraction (81.3 mg RE/g) was reported in the flavonoid fraction compared with the phenolic one (36.2 µg GAE/g), indicating that AMRE is particularly rich in flavonoids as a major class of polyphenols among its composition Fig. 2.

### Analytical validation and reliability

**Fig. 3:**
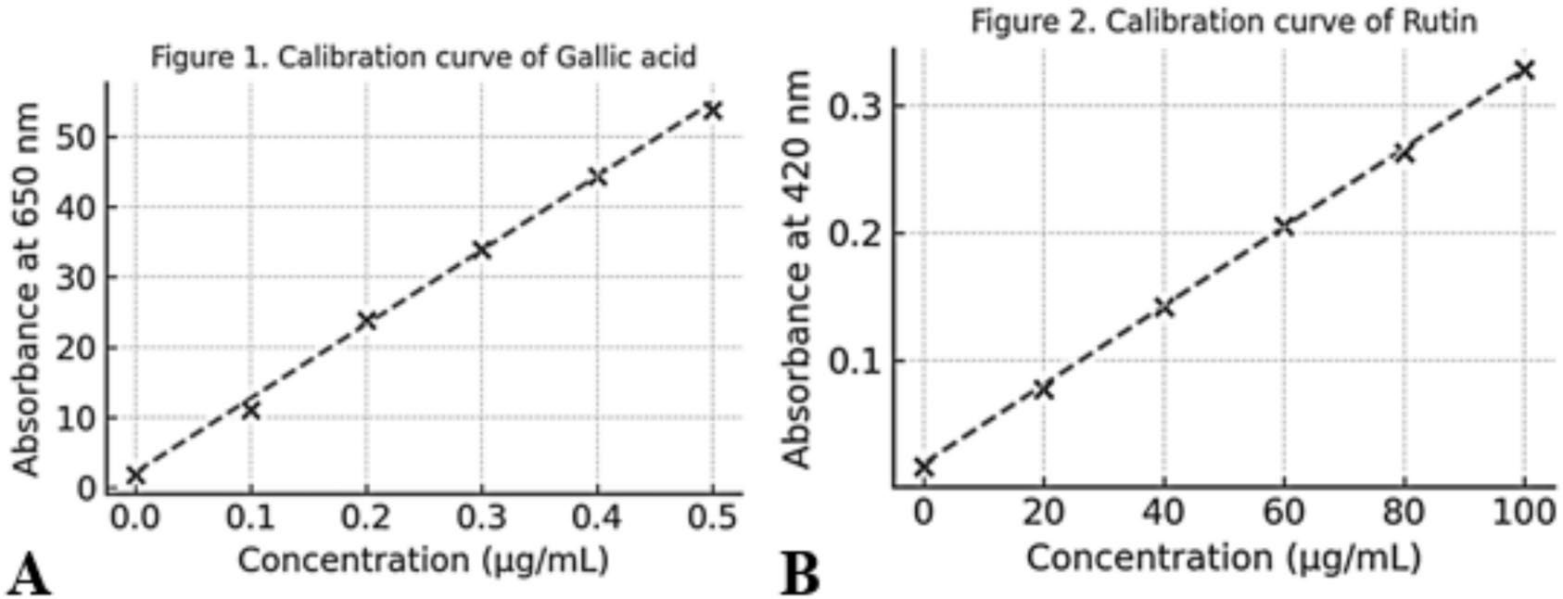
(**A**) Calibration curve of gallic acid standard used for the determination of total phenolic contents in AMRE (Folin–Ciocalteu method). Strong correlation between Gallic acid concentration and absorbance is expressed as the linear regression (y = 105.51x + 2.1824, R^2^ = 0.9978). (**B**) Calibration curve of standard rutin in the determination of total content of flavonoid in AMRE (AlCl_3_ colorimetric method). A linear relationship (y = 0.0031x + 0.0188, R^2^ = 0.9959) is obtained, suggesting excellent analytical linearity.

The linear regression equation derived from the calibration curves of gallic acid (for TPC) and rutin (for TFC) revealed a good analytical linearity with R^2^ values exceeding 0.99 as represented in Fig. 3. The intercept values represent the zero-concentration absorbance. R^2^ values (0.9978 for phenolics and 0.9959 for flavonoids) demonstrate that over 99% of the absorbance data variation can be explained directly by changes in concentration and indicate the reliability and repeatability of both spectrophotometric assays used. Together, these criteria demonstrate that the proposed methods are accurate for quantitative analysis of AMRE.

### Physicochemical characterization of aluminum oxide nanoparticles

**Fig. 4:**
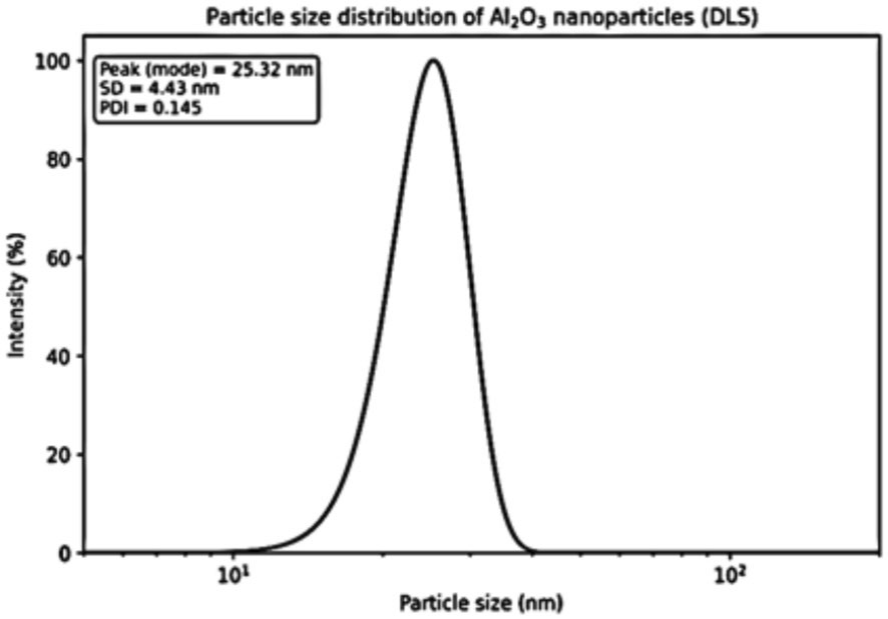
Size distribution profile of Al_2_O_3_-NPs as determined by dynamic light scattering (DLS).

The physicochemical properties of Al_2_O_3_ nanoparticles were systematically examined to verify their nanoscale nature and to establish a mechanistic relationship with the observed toxicological effects in Nile tilapia. Dynamic light scattering (DLS) analysis revealed a narrow and monodispersed particle size distribution of Al_2_O_3_-NPs with mean hydrodynamic diameters ranging between 25–30 nm, and DIs = 0.145, suggesting high homogeneity in composition and particle size (Fig. 4). The nanometric size and narrow distribution are of particular importance in aquatic nanotoxicology due to their capability to maintain a stable exposure scenario, promoting nanoparticle mobility and biological accessibility, particularly through gill epithelium as one of major pathways for nanoparticles uptake in fish.

**Fig. 5:**
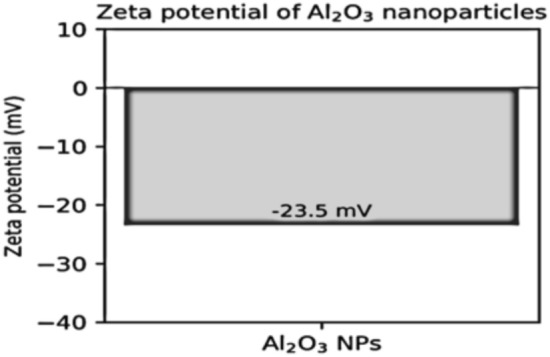
Zeta potential of Al_2_O_3_-NPs as determined by dynamic light scattering (DLS).

In addition to particle size, the ZP of Al_2_O_3_-NPs was determined by zeta potential measurements. These nanoparticles with a relatively negative zeta potential (~ − 23.5 mV) indicating that the colloidal suspension could be well electrostatically stabilized, meanwhile, some aggregations were allowed in water ionic environment due to the charge screening (Fig. 5). This mid-range colloidal stability is ideal for aquatic nanotoxicology as it prevents rapid sedimentation processes while maintaining effective contact with negatively charged biological membranes and mucus layers thereby enhancing nanoparticles bioavailability and subsequent toxicological responses.

**Fig. 6:**
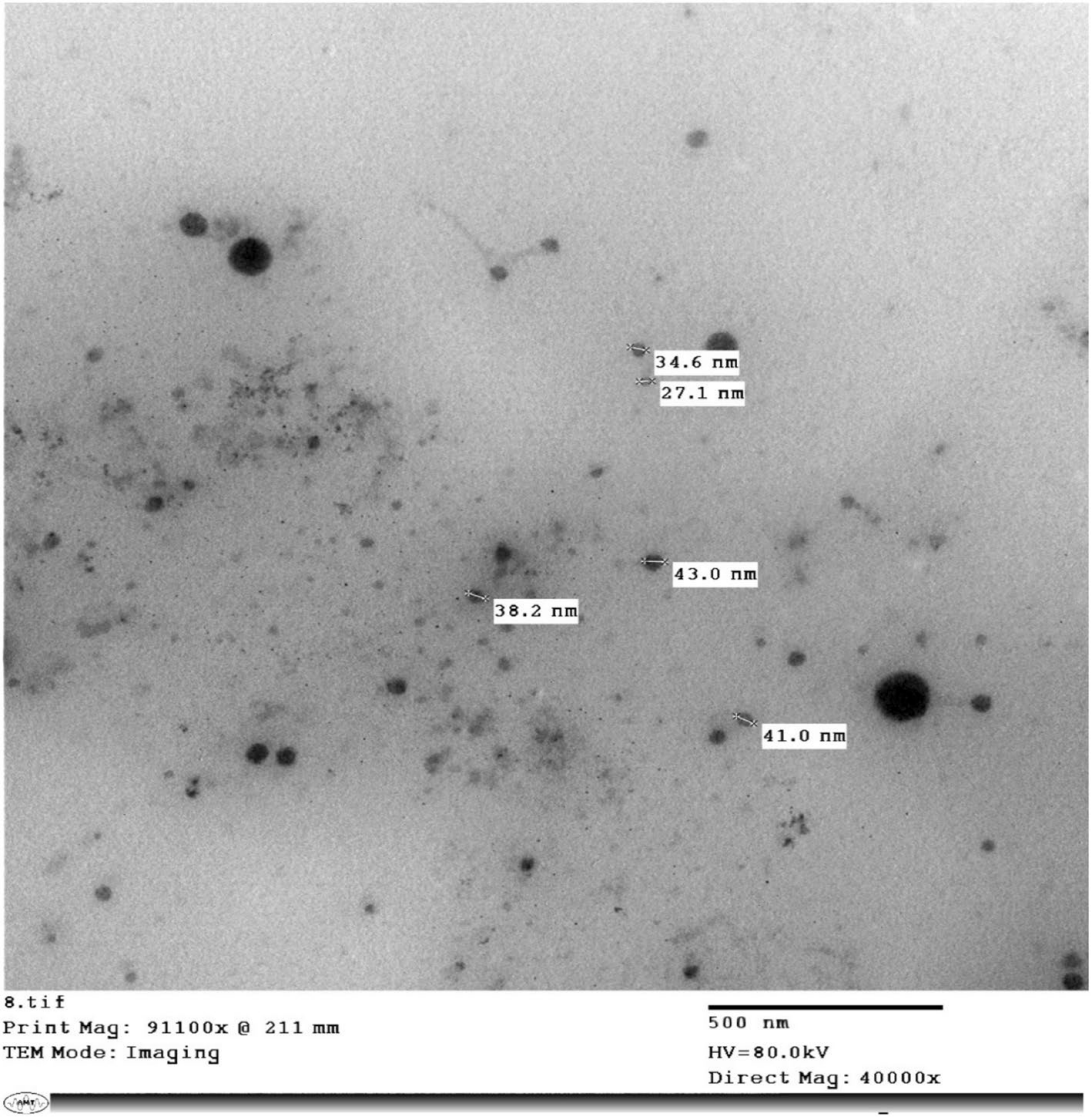
Transmission electron microscope (TEM) picture of Al_2_O_3_-NPs revealing mostly spherical dispersed particles in the size range of ∼25–40 nm. The reduced aggregation and homogeneous nanoscale morphology validate the DLS measurements and are consistent with the physicochemical mechanism of particle-induced toxicity.

The morphology and size of the Al_2_O_3_-NPs was also characterized using Transmission Electron Microscopy (TEM). TEM micrographs showed mainly spherical, dispersed nanoparticles with particle size ranging from 25 to 40 nm and there was minimal agglomeration (Fig. 6). The average sizes of the particles estimated from TEM were slightly smaller than those determined by DLS, as expected due to the contribution of solvation layers and Brownian motion in light scattering methods. The observed morphological homogeneity and limited aggregation further confirm DLS and zeta potential data.

As a whole, the combined DLS, zeta potential and TEM results revealed that Al_2_O_3_-NPs possess small size, narrow particle size distribution, moderate surface charge and uniform nanoscale morphology. These physicochemical properties are strong evidence for the enhanced bioavailability and biological reactivity of these nanoparticles, which enables them to exert toxic effects on Nile tilapia following aqueous exposure.

### Clinical symptoms of treated fish

**Fig. 7:**
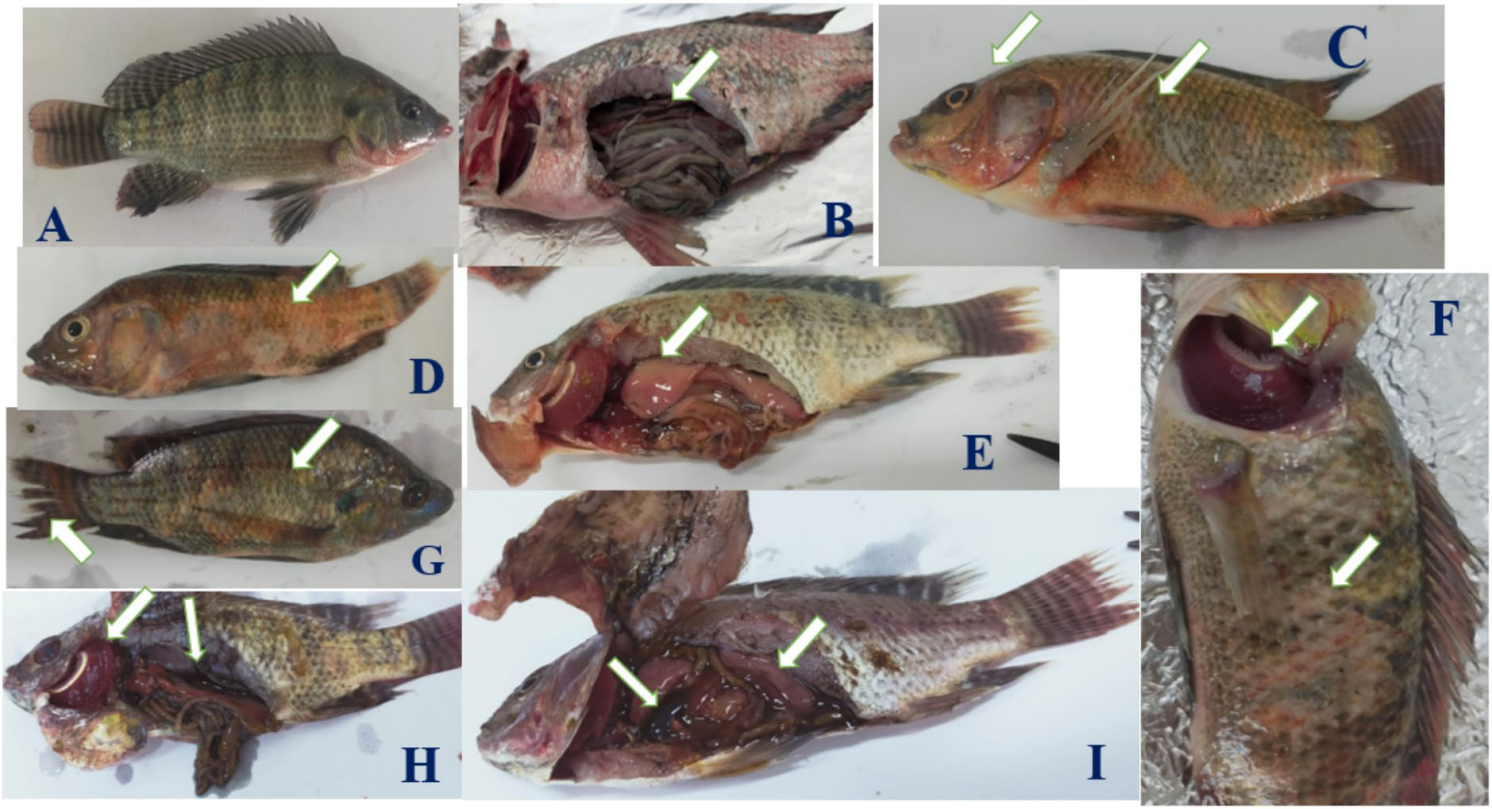
Clinical symptoms of treated fish. Apparently normal fish in groups (1, 2, 3, 5, and 6) showed normal gills, skin, and internal organs (A, B). Groups exposed to Al_2_O_3_-NPs in group (4) showed external severe loss of scales, hemorrhagic patches on skin, tail rot, congested gills, hemorrhages in the eye and skin darkness. Internally, there was an enlarged congested liver, accumulation of discharges in the abdomen, and an engorged gall bladder (C-I).

Throughout the experimental period, fish in 1, 2, 3, 5, and 6 groups appeared clinically normal without observable external lesions Fig. 7A,B. Fish in group (4) exposed to Al_2_O_3_-NPs exhibited severe external loss of scales, hemorrhagic patches on skin, tail rot, ocular hemorrhage and skin darkness Fig. 7C,D,F,G,H. Internally, there was an enlarged congested liver, accumulation of serous exudates in the abdomen, and an engorged gall bladder Fig. 7E,H,I.

### Growth performance, feed utilization, and fish survivability

As represented in Table 5 exposure to Al_2_O_3_-NPs revealed significant decreases (*P* < 0.05) in final weight (g), weight gain (g), weight gain rate %, and specific growth rate versus the control group. Also, survivability showed a significant decrease (*P* < 0.05) versus the control group. FCR deteriorated versus the control group. Fish groups co-treated with APS showed a significant improvement (*P* < 0.05) in growth parameters as final weight (g), weight gain (g), and weight gain rate (%) versus Al_2_O_3_-NPs-exposed group. FCR improved in compared with the Al_2_O_3_-NPs-exposed group. Co-treated groups exhibited improved survivability versus the Al_2_O_3_-NPs-exposed group.

**Table 5:** The effects of Aluminum oxide nanoparticles (Al_2_O_3_NPs) and/or APS on growth performance and survival rates of *O. niloticus* fish during the 4-weeks feeding trial.

Fish groups	Growth performance values							
	Initial weights (g/Fish)	Final weights (g/Fish)	Weight gains (g/fish)	Weight gain rate (%)	Feed intake (g/fish)	Feed conversion rate	Specific growth rate (%/D)	Survivability (%)
Control	25.28 ± 0.08 ^a^	45.20 ± 0.13^d^	19.92 ± 0.08^d^	78.80 ± 0.40^d^	33.20 ± 0.58^c^	1.66 ± 0.03^bc^	1.93 ± 0.005^d^	98%^a^
APS 1.5	25.28 ± 0.08 ^a^	7.60 ± 0.17^c^	22.32 ± 0.15^c^	88.29 ± 0.71^c^	36.50 ± 0.41^b^	1.63 ± 0.02^bc^	2.10 ± 0.01^c^	100%^a^
APS 3	25.28 ± 0.08 ^a^	50.86 ± 0.45^a^	25.58 ± 0.42^a^	101.18 ± 1.62^a^	41.20 ± 0.46^a^	1.61 ± 0.01^c^	2.32 ± 0.02^a^	100%^a^
Al_2_O_3_-NPs	25.28 ± 0.08^a^	40.94 ± 0.37^e^	15.66 ± 0.30^e^	61.93 ± 1.04^e^	30.42 ± 0.28^d^	1.94 ± 0.02^a^	1.60 ± 0.02^e^	95%^b^
APS 1.5 + Al_2_O_3_-NPs	25.28 ± 0.08 ^a^	46.66 ± 0.26^c^	21.38 ± 0.28^c^	84.58 ± 1.23^c^	37.30 ± 0.80^b^	1.74 ± 0.03^b^	2.04 ± 0.02^c^	100%^a^
APS 3 + Al_2_O_3_-NPs	25.28 ± 0.08 ^a^	49.26 ± 0.29^b^	23.98 ± 0.31^b^	94.86 ± 1.39^b^	40.28 ± 0.20^a^	1.68 ± 0.02^bc^	2.22 ± 0.02^b^	100%^a^
*P* -value	1.000	< 0.001	< 0.001	< 0.001	< 0.001	< 0.001	< 0.001	< 0.001

Values are represented as the mean ± standard error of the mean (SEM) for five fish per replicate (*n* = 5). Significant differences (*P* < 0.05) between groups within the same column are denoted by different lower-case letters.

### Water quality analysis

Throughout the experimental period, all water quality parameters remained within the PL (permissible limits), except for NO_2_ levels, which exceeded the PL in all groups according to EPA^50^. Significant difference in TAN, NO_2_, and NO_3_ concentrations were observed between all groups (*P* < 0.05) as shown in Table 6.

**Table 6:** The effect of Al_2_O_3_NPs and/or APS on water quality parameters during the 4-weeks feeding trial.

Groups	Water quality parameters						
	Temperature	Dissolved oxygen (mg/L)	PH	Electrical conductivity (μS/cm)	Total ammonia nitrogen (mg/L)	NO3 (mg/L)	NO_2_ (mg/L)
Control	29.08 ± 0.11	6.66 ± 0.21	6.60 ± 0.16	60.06 ± 0.24	0.0012 ± 0.00^b^	0.061 ± 0.00^b^	0.183 ± 0.00^a^
APS 1.5	28.80 ± 0.32	6.64 ± 0.20	6.76 ± 0.07	59.44 ± 0.16	0.0012 ± 0.00^b^	0.061 ± 0.00^b^	0.183 ± 0.00^a^
APS3	28.90 ± 0.43	6.72 ± 0.20	6.78 ± 0.05	59.54 ± 0.12	0.0012 ± 0.00^b^	0.061 ± 0.00^b^	0.183 ± 0.00^a^
Al_2_O_3-_NPs	28.70 ± 0.26	6.34 ± 0.28	6.76 ± 0.07	60.12 ± 0.22	0.0018 ± 0.00^a^	0.065 ± 0.00^a^	0.176 ± 0.00^b^
APS 1.5 + Al_2_O_3-_NPs	28.90 ± 0.41	6.88 ± 0.03	6.82 ± 0.08	59.54 ± 0.16	0.0012 ± 0.00^b^	0.061 ± 0.00^b^	0.184 ± 0.00^a^
APS 3 + Al_2_O_3-_NPs	28.90 ± 0.28	6.70 ± 0.20	6.66 ± 0.08	59.92 ± 0.29	0.0012 ± 0.00^b^	0.061 ± 0.00^b^	0.183 ± 0.00^a^
*P* -value	0.976	0.597	0.602	0.118	< 0.001	< 0.001	< 0.001

Values are represented as the mean ± standard error of the mean (SEM) (*n* = 5/replicate). Significant differences (*P* < 0.05) between groups within the same column are denoted by different lower-case letters.

### Biochemical and immunological parameters analysis

As displayed in Table 7 fish group exposed to Al_2_O_3_-NPs exhibited liver dysfunction marked by a significant increase (*P* < 0.05) in serum alanine aminotransferase (ALT) activity and significant decrease (*P* < 0.05) in total protein versus the control group. Moreover, serum immunoglobulin M (IgM) concentration was significantly decreased (*P* < 0.05) compared to the control group. Co-treatment with APS (especially high dose) revealed a marked reduction in ALT and a concomitant elevation in total protein levels versus the Al_2_O_3_-NPs-exposed group. Similarly, IgM levels were significantly increased (*P* < 0.05) in fish groups co-treated with APS (1.5–3 mg /kg diet) versus the Al_2_O_3_-NPs-exposed group.

**Table 7:** Biochemical and immunological parameters analysis of fish groups during the 4-week feeding trial.

Groups	Biochemical and immunological parameters					
	ALT (U/L)	TP (g/dL)	ALB (g/dL)	GLO (g/dL)	A/G Ratio	IgM (mg/dL)
Control	6.14 ± 0.01^b^	3.40 ± 0.01 ^a^	2.04 ± 0.01 ^a^	1.36 ± 0.01 ^c^	1.50 ± 0.01 ^a^	2.38 ± 0.01 ^b^
APS 1.5	5.40 ± 0.05 ^c^	3.48 ± 0.04 ^a^	1.65 ± 0.05 ^b^	1.83 ± 0.05 ^a^	0.90 ± 0.03 ^b^	2.50 ± 0.05 ^a^
APS 3	3.60 ± 0.04 ^d^	3.23 ± 0.04 ^a^	1.48 ± 0.04 ^b^	1.75 ± 0.04 ^a^	0.85 ± 0.03 ^b^	2.22 ± 0.04 ^b^
Al_2_O_3-_NPs	8.53 ± 0.01 ^a^	2.50 ± 0.01 ^b^	0.90 ± 0.01 ^c^	1.60 ± 0.01 ^b^	0.56 ± 0.01 ^c^	1.92 ± 0.01 ^c^
APS 1.5 + Al_2_O_3-_NPs	4.64 ± 0.06 cd	3.25 ± 0.04 ^a^	1.52 ± 0.05 ^b^	1.73 ± 0.04 ^a^	0.88 ± 0.03 ^b^	1.75 ± 0.05 ^c^
APS 3 + Al_2_O_3-_NPs	3.40 ± 0.05 ^d^	3.24 ± 0.04 ^a^	1.43 ± 0.05 ^b^	1.81 ± 0.05 ^a^	0.79 ± 0.03 ^b^	2.09 ± 0.04 ^b^
*P-*value	< 0.001	< 0.001	< 0.001	< 0.001	< 0.001	< 0.001

Values are represented as the mean ± standard error of the mean (SEM) for five fish per replicate (*n* = 5). Significant differences (*P* < 0.05) between groups within the same column are denoted by different lower-case letters. Similar letters indicate no significant differences.

### Real-time PCR analysis

#### mRNA relative expression of antioxidant-related genes (*CAT *and *SOD*)

**Fig. 8:**
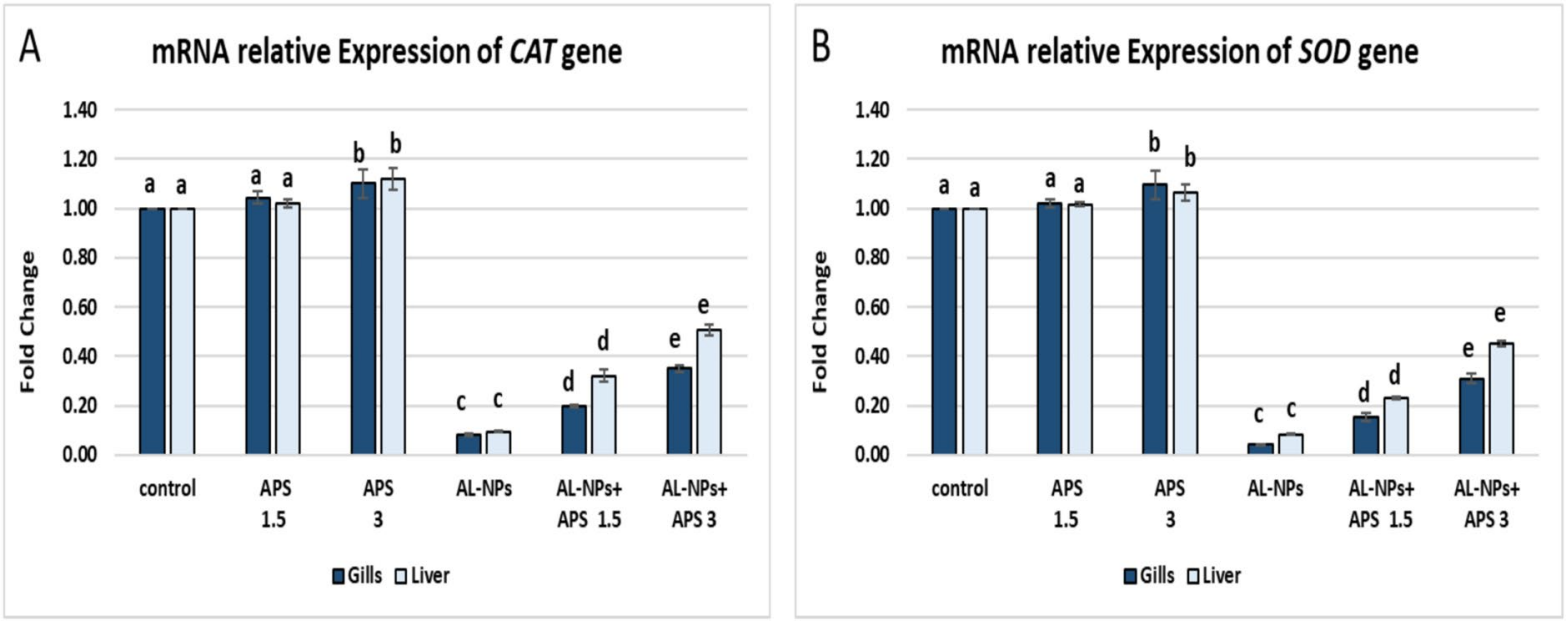
The effect of Al_2_O_3_-NPs and/or APS on mRNA relative expression of the *CAT* gene (**A**) the *SOD* gene (**B**) in the gills, and the liver of *O. Niloticus*. Data are represented as mean ± SEM (*n* = 5/replicate). Groups having different letters are significantly different from each other at *P* < 0.05. Groups having similar letters are non-significantly different from each other at *P* < 0.05.

As illustrated in Fig. 8 fish group exposed to Al_2_O_3_-NPs showed notably down-regulation (*P* < 0.05) in the *CAT* gene expression in the gills and liver (0.08 and 0.10-fold respectively) in relation to the control group. Similarly, *SOD* gene expression was significantly down-regulated (*P* < 0.05) in the gills and liver to 0.05 and 0.08-fold respectively compared with the control one. Fish groups co-treated with APS showed significant up-regulation in the expression level of both genes in comparison with the Al_2_O_3_-NPs-exposed group (*P* < 0.05). The co-treatment with the low dose of APS (1.5 mg/kg diet) significantly upregulated (*P* < 0.05) *CAT* expression in the gills and liver to 0.2 and 0.32-fold respectively. Furthermore, the enhancement in the expression of *CAT* gene by the high dose of APS (3 mg/kg diet) was 0.35 and 0.51-fold in the gills and liver, respectively, compared with the Al_2_O_3_-NPs-exposed group. The downregulation of *SOD* expression by the co-treatment with the low dose of APS was 0.15 and 0.23-fold in the gills and liver, respectively. In addition, the high dose of APS co-treatment significantly upregulated (*P* < 0.05) the expression of the gene in the gills and liver to 0.32 and 0.45-fold respectively compared with the Al_2_O_3_-NPs-exposed group.

#### mRNA relative expression of immune related genes (*TNF-β* and *IL-1β*)

**Fig. 9:**
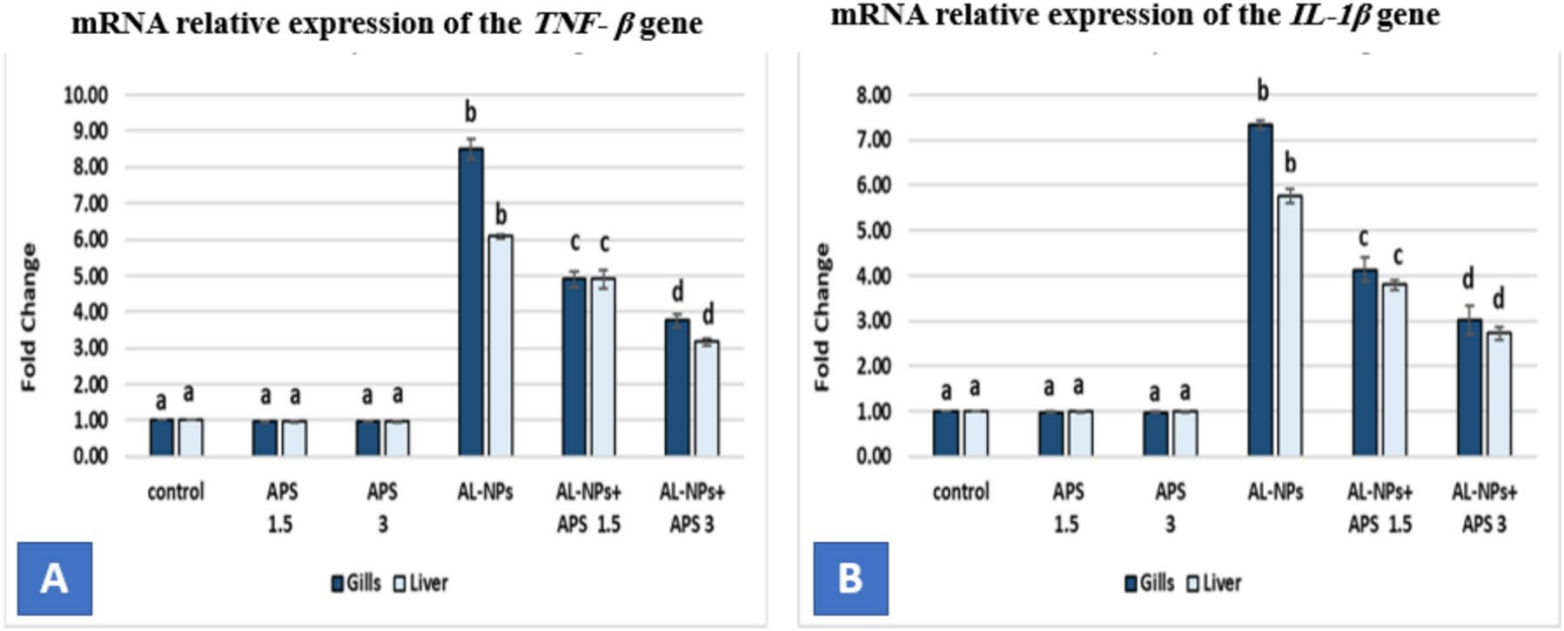
The effect of Al_2_O_3_-NPs and/or APS on mRNA relative expression of the *TNF- β* gene (**A**) the *IL-1β* gene (**B**) in the gills, and liver of *O. Niloticus*. Data are represented as mean ± SEM (*n* = 5/replicate). Groups having different letters are significantly different from each other at *P* < 0.05. Groups having similar letters are non-significantly different from each other at *P* < 0.05.

As shown in Fig. 9 fish group exposed to Al_2_O_3_-NPs represented significant up-regulation of *TNF-β* expression in the gills, and liver to 8.50 and 6.09-fold respectively compared with the control group (*P* < 0.05). On the same line, the expression of *IL-1β* gene in the gills and liver was significantly up-regulated (*P* < 0.05) to 7.34 and 5.77-fold respectively versus the control group. Co-treatment with APS significantly ameliorated the expression levels of both genes in comparison with the Al_2_O_3_-NPs-exposed group (*P* < 0.05). The co-treatment with the low dose of APS (1.5 mg/kg diet) significantly downregulated (*P* < 0.05) the expression of *TNF-β* in the gills and liver to 4.13 and 3.80-fold respectively. The improvement in the expression of the *TNF-β* gene achieved by the high dose of APS (3 mg/kg diet) was 3.03 and 2.73-fold in the gills and liver, respectively, compared with the Al_2_O_3_-NPs-exposed group (*P* < 0.05). The downregulation of *IL-1β* expression by co-treatment with the low dose of APS was 4.13 and 3.80-fold in the gills and liver respectively. Additionally, the high dose of APS co-treatment significantly downregulated the expression of the gene in the gills and liver to 3.03 and 2.73-fold respectively in comparison with the Al_2_O_3_-NPs-exposed group (*P* < 0.05).

#### mRNA relative expression of the *MT* gene

**Fig. 10:**
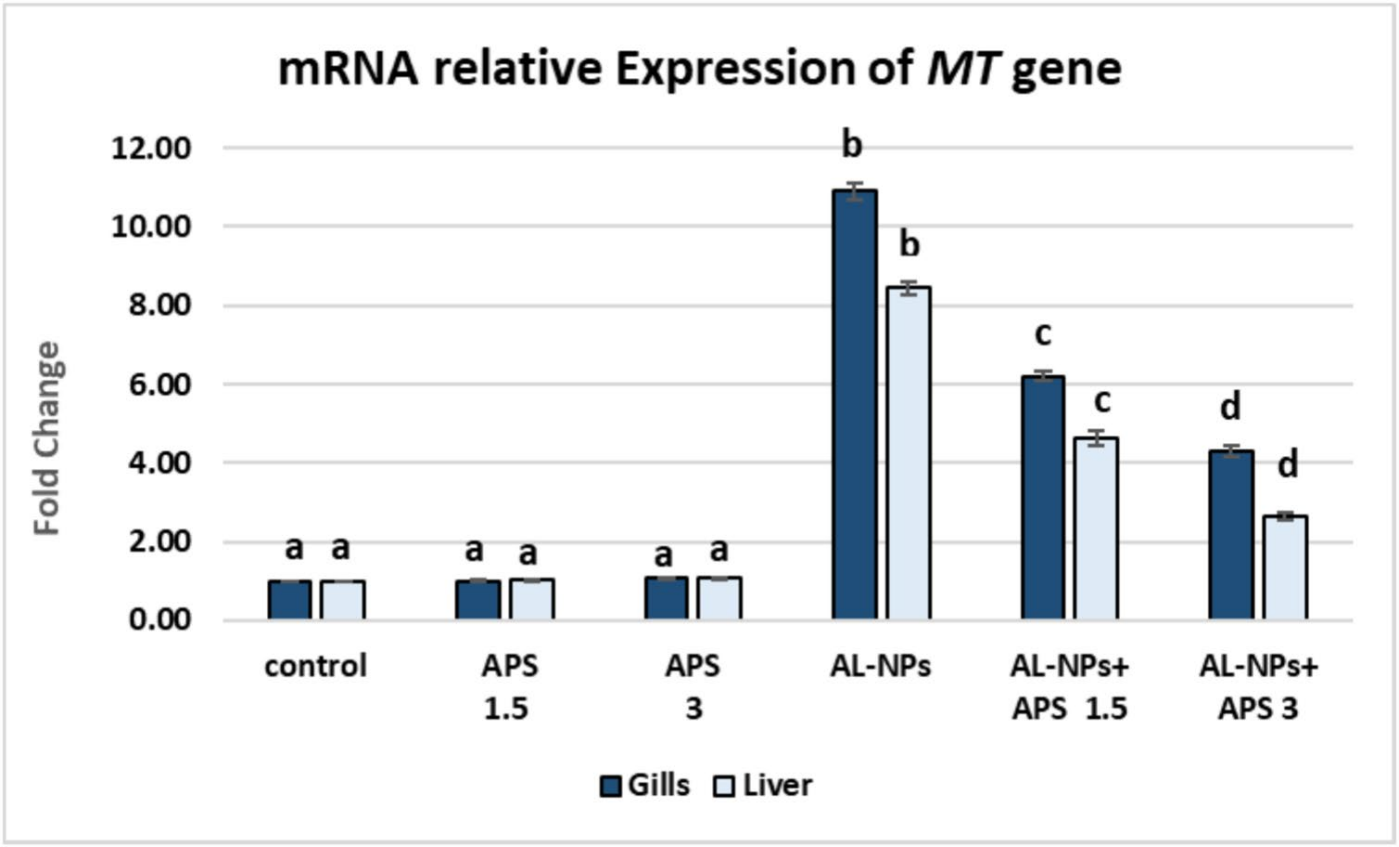
The effect of Al_2_O_3_-NPs and/or APS on mRNA relative expression of the *MT* gene in the gills, and liver of *O. Niloticus*. Data are represented as mean ± SEM (*n* = 5/replicate). Groups having different letters are significantly different from each other at *P* < 0.05. Groups having similar letters are non-significantly different from each other at *P* < 0.05.

As illustrated in Fig. 10, fish group exposed to Al_2_O_3_-NPs exhibited significant up-regulation of *MT* expression in both gills and liver to 10.90 and 8.43-fold, respectively, compared with the control group (*P* < 0.05). Co-treatment with APS significantly down-regulated *MT* expression in both gills and liver in comparison with the Al_2_O_3_-NPs-exposed group (*P* < 0.05). The co-treatment with low dose of APS (1.5 mg/kg diet) downregulated *MT* expression to 6.20 and 4.63-fold in the gills and liver, respectively. Furthermore, the co-treatment with high dose of APS (3 mg/kg diet) notably downregulated the expression of *MT* in the gills and liver to 4.30 and 2.63-fold, respectively in comparison with the Al_2_O_3_-NPs-exposed group (*P* < 0.05).

### Histopathological examination

#### Gills

**Fig. 11:**
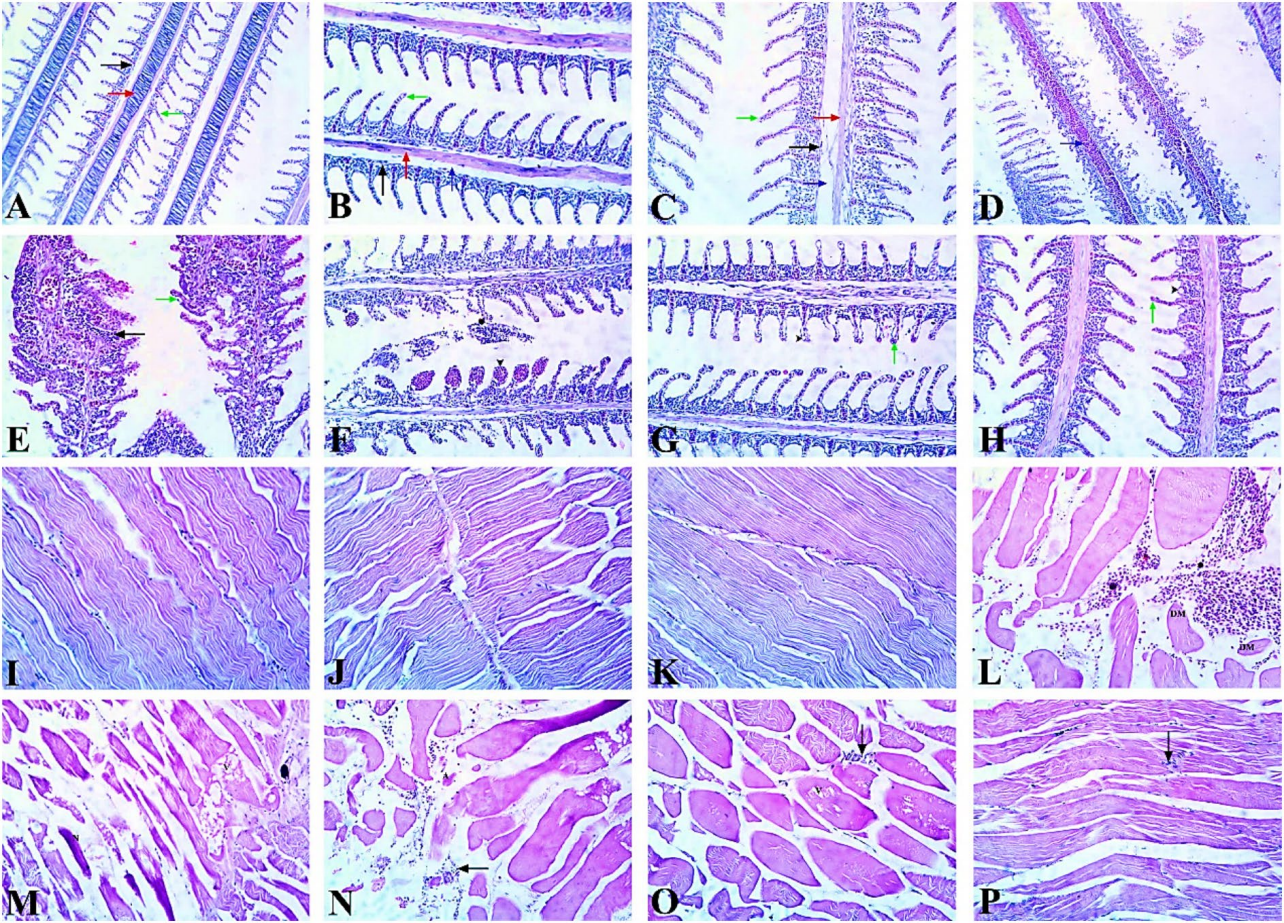
Representative photomicrograph of H&E-stained gills (**A**–**H**) and muscle (**I**–**P**) sections of Nile Tilapia fish. (**A**–**C**) Control and APS treated groups are showing normal histological structure of primary lamellae (black arrow), secondary lamellae (green arrow), central cartilaginous support (red arrow), and central venous sinus (blue arrow). (**D**–**F**) The Al_2_O_3_-NPs group is showing marked congestion and dilatation of the central venous sinus (blue arrow), fusion of secondary lamellae (green arrow), epithelial cell hyperplasia and hypertrophy, massive inflammatory cell infiltration (black arrow) and aneurysm (arrowhead) of the lamellar vascular axis of secondary lamellae. Note sloughed necrotic cells mixed with cellular debris (asterisk) at the gill surface. (**G**) The low-dose co-treated APS group (1.5) is showing mild oedema, congestion of the lamellar vascular axis (green arrow), epithelial degeneration and lifting (arrowhead). (**H**) The high-dose APS co-treated group showing mild hyperplasia of epithelial cells (arrowhead) and congestion of the lamellar vascular axis (green arrow). (**I**–**K**) Muscles of control and APS-treated groups are showing normal striation. (**L**–**N**) Al_2_O_3_NPs-exposed group is showing widespread hemorrhages (asterisk) and interstitial oedema between degenerated muscles (DM). The muscular degenerative alterations include; myocyte vacuolation (**V**), absence of normal muscle striation, Zenker’s necrosis (N), mononuclear inflammatory cell infiltration (black arrow) and muscular atrophy (**A**). (**O**) The low-dose APS co-treated group (1.5) is showing mild myocyte vacuolation (V), leukocytic cellular infiltration (black arrow). (**P**) The high-dose APS co-treated group (3) is showing nearly normal muscle tissue with minimal leukocytic cellular infiltration (black arrow).

Microscopic examination of gills in the control and APS-treated groups revealed normal histological appearance where the primary and secondary lamellae, central cartilaginous support, and the central venous sinus were well-structured. However, the gills exposed to Al_2_O_3_-NPs exhibited severe histopathological alterations, characterized by marked congestion and dilatation of the central venous sinus in primary lamellae. The secondary gill lamellae showed extensive fusion with diffuse epithelial cell hyperplasia and hypertrophy, multifocal hyperplasia and hypertrophy of mucous cells. Massive inflammatory cell infiltration and occasional aneurysm of the lamellar vascular axis were also observed. Conversely, the groups co-treated with both low and high doses of APS (1.5 and 3 mg/kg diet respectively) showed a noticeable improvement, and the severity of the majority of degenerative alterations were decreased. The low-dose APS co-treated group (1.5 mg/kg diet) exhibited nearly regular, well-structured primary and secondary lamellae, as well as central cartilaginous support. However, some degenerative changes were also identified, but with less severity compared to the Al_2_O_3_-NPs-exposed group, such as mild edema, moderate dilatation, congestion of the lamellar vascular axis, epithelial degeneration and lifting. Meanwhile, the co-treated group with high-dose APS (3 mg/kg diet) significantly reduced the damage and restored the cytoarchitecture of the gills. Most sections revealed the normal histological structure of gills with only minor degenerative changes, such as mild hyperplasia of epithelial cells and congestion of the lamellar vascular axis Fig. 11A–H.

#### Muscles

Microscopic examination of the muscles in both the control and APS-treated group showed normal striation with no observable histological alterations. Meanwhile, muscles from the Al_2_O_3_-NPs exposed group revealed marked histological alterations, including widespread hemorrhages and interstitial oedema between the degenerated muscle fibers. The muscular degenerative alterations were characterized by significant myocyte vacuolation and the absence of normal muscle striation. Zenker’s necrosis, characterized by hyper-eosinophilic sarcoplasm, myocyte fading, and mononuclear inflammatory cell infiltration, was also frequently detected. Muscular atrophy, indicated by a significant decrease in the size of muscle fibers, was particularly noticeable. Furthermore, APS co-treatment retains muscle striation and restores it to a virtually normal histological appearance. The low-dose APS co-treated group (1.5 mg/kg diet) reduced the degenerative muscle alterations caused by Al_2_O_3_-NPs to some extent, with the exception of mild myocyte vacuolation, leukocytic cellular infiltration, and intercellular oedema. The high-dose APS co-treated group (3 mg/kg diet) demonstrated a greater protective effect against muscle injury caused by Al_2_O_3_-NPs exposure. The muscle tissue appeared normal, with minimal leukocytic cellular infiltration (Fig. 11I–P).

#### Liver

**Fig. 12:**
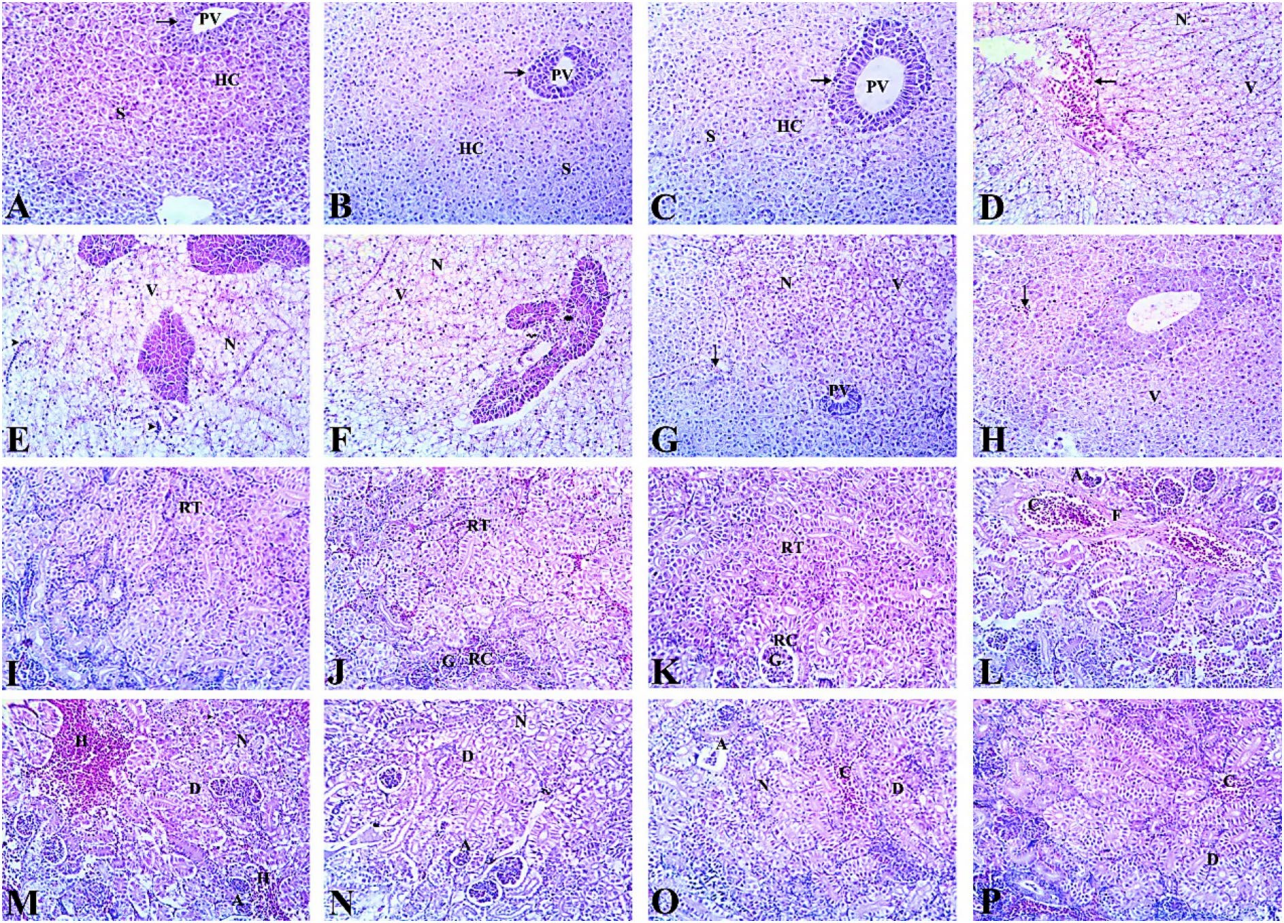
Representative photomicrograph of H&E-stained (× 200) liver (**A**–**H**) and kidney (**I**–**P**) sections of fish. (**A**–**C**) Control and APS supplemented groups showing normal histological appearance of liver tissues; polygonal hepatocytes (HC), narrow blood sinusoids (S), and hepatopancreas (arrow) surrounding the portal vein (PV). (**D**–**F**) Al_2_O_3_NPs-exposed group is showing extensive vascular congestion (arrow), inflammatory cell infiltrations (arrowhead), hepatocytes cytoplasmic vacuolization (V), and necrosis (N). Note also degenerative changes in hepatopancreas (asterisk) with infiltration of inflammatory cells (zigzag arrow). (G) The low dose APS co-treated group (1.5) is showing mild congestion of the blood sinusoid (arrow) and the portal vein (PV) along with moderate vacuolation (V) and necrosis (N) of hepatocytes. (H) The high dose APS co-treated group (3) is showing mild blood congestion (arrow) and cellular degeneration with cytoplasmic vacuolization (V). (**I**–**K**) Kidneys of the control and APS-treated groups are showing normal renal tubules (RT), renal corpuscles (RC) with well-developed glomeruli (**G**). (**L**–**N**) The Al_2_O_3_NPs exposed group is showing severe congestion (C) of renal blood vessels, as well as widespread fibrosis (F) in the vascular walls; multifocal haemorrhages along with scattered melanomacrophages (arrowhead) in interstitial renal tissue. The renal tubules are showing degeneration (D) and necrosis (N) of their lining epithelial cells. The glomerulus is showing congestion, atrophy, and a widened space between the glomerulus and Bowman’s capsule. (**O**) The low dose APS co-treated group (1.5) is showing moderate congestion (C) with moderate degeneration (D) and necrosis (N) of renal tubules, note also atrophy (A) in some glomeruli. (P) The high dose APS co-treated group (3) is showing mild blood congestion (C) and cellular degeneration (D).

Microscopic examination of the liver in control and APS-treated groups showed normal architecture of liver tissues, including polygonal hepatocytes (central vesicular nuclei and homogenous cytoplasm), and narrow blood sinusoids were distributed randomly all over the hepatic parenchyma separating the hepatocytes. Concerning the hepatopancreas, it had a normal structure as islets with their lamellar lining arranged in an acinar pattern encircling a branch of the portal vein among the hepatocytes. On the other hand, the Al_2_O_3_-NPs-exposed group reveled severe histopathological changes including blood congestion (the central vein, blood sinusoids), severe cellular degeneration, and inflammatory cell infiltrations. The hepatocytes showed swelling with severe cytoplasmic vacuolization and eccentric, darkly stained nuclei. Additionally, focal hepatocyte necrosis with pyknotic nuclei was noticed in most examined liver sections. Concerning the hepatopancreas of the Al_2_O_3_-NPs-exposed group, it showed irregular walls, necrosis of their acini and infiltration of inflammatory cells. Moreover, the effects of Al_2_O_3_-NPs exposure were somewhat recovered by co-treatment with APS where the low dose APS co-treated group (1.5 mg/kg diet) showed mild congestion of hepatic sinusoids and portal blood vessels together with degeneration and necrosis of some hepatocytes. However, the high dose APS co-treated group (3 mg/kg diet) markedly mitigated the liver injury induced by Al_2_O_3_-NPs. The cytoarchitecture of hepatic parenchyma was restored to nearly normal appearance except for mild blood congestion and cellular degeneration with cytoplasmic vacuolization (Fig. 12A–H).

#### Kidney

Microscopic examination of kidneys in both control and APS-treated groups revealed a normal histological architecture, characterized by regularly formed tubules and numerous renal corpuscles with well-developed glomeruli. Whereas, Al_2_O_3_-NPs exposed group displayed severe congestion and dilatation of renal blood vessels as well as widespread fibrosis in their walls. Multifocal hemorrhages along with scattered melanomacrophages in interstitial renal tissue were also noticed. The lining epithelial cells of the renal tubules had necrosis and degeneration, along with congested glomeruli, glomerular shrinkage, and widened space between the glomerulus and Bowman’s capsule. The histological picture of the kidneys in APS co-treated groups was relatively better than in the Al_2_O_3_-NPs-exposed group, where the low-dose APS co-treated group (1.5 mg/kg diet) resulted in moderate congestion and degenerative changes in renal architecture. The high-dose APS co-treated group (3 mg/kg diet) resulted in a marked restoration of renal parenchymal cytoarchitecture to a virtually normal look, with the exception of mild blood congestion and cellular degeneration (Fig. 12I–P).

#### Spleen

**Fig. 13:**
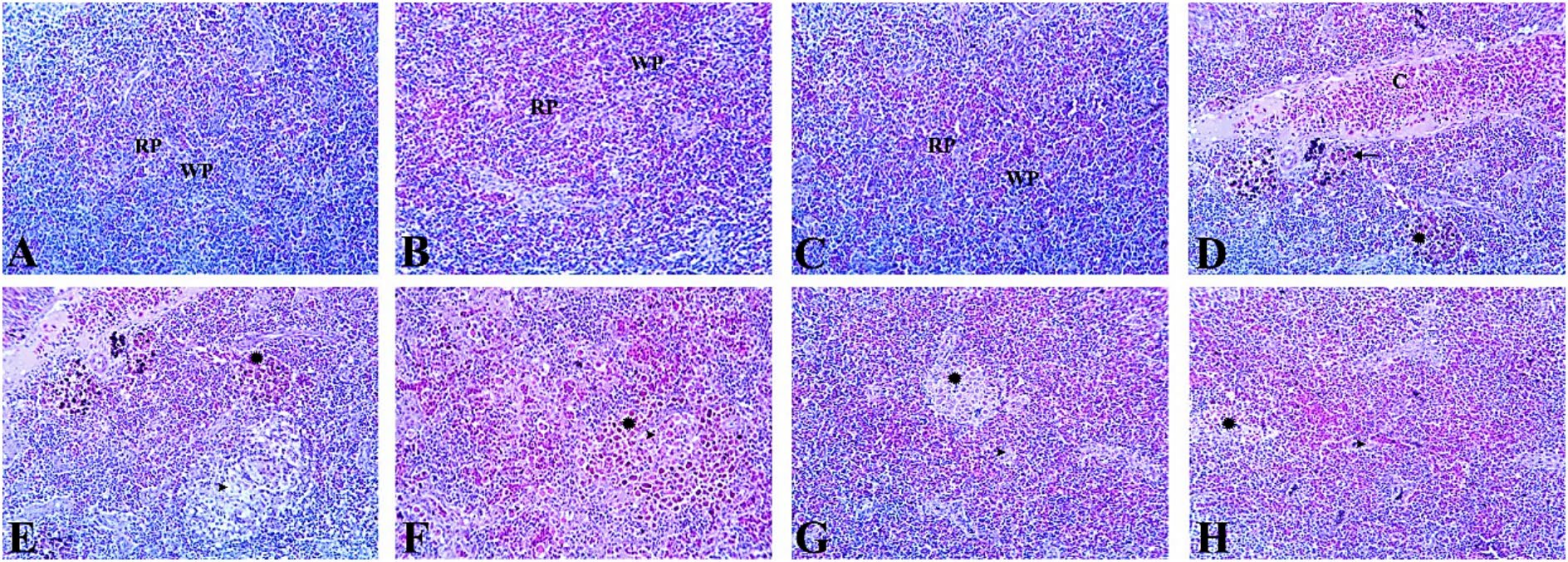
Representative photomicrograph of H&E-stained (× 200) spleen sections of fish. (**A**–**C**) The Control and the APS-treated groups are showing normal appearance of white pulp (WP) and red pulp (RP). (**D**–**F**) The Al_2_O_3_NPs-exposed group is showing extensive congestion (C) and dilatation of splenic blood vessels, hyperactivation of many brown melanomacrophages clusters around blood vessels (arrow), and distributed within splenic parenchyma (asterisk). Also noted lymphocytolysis and depletion of lymphoid follicles (arrowhead). (**G**) The low-dose APS co-treated group (1.5) is showing mild deposits of hemosiderin pigment (asterisk) with a moderate degree of lymphoid depletion (arrowhead). (**H**) The high-dose APS co-treated group (3) is showing low-density melanomacrophage centers (asterisk) and a mild degree of lymphoid depletion (arrowhead).

The spleen of both the control and APS-treated groups microscopically consists of intermixed white pulp and red pulp. In the Al_2_O_3_-NPs-exposed group, the spleen showed marked distortion of splenic architecture represented in extensive congestion and dilatation of splenic blood vessels. Many brown melanomacrophages clusters, mainly around blood vessels, were hyperactivated, with disseminated dark-brown hemosiderin deposits disrupting large areas of splenic parenchyma. Lymphocytolysis and depletion of lymphoid follicles were common finding in this group. The supplementation of APS with Al_2_O_3_-NPs resulted in the restoration of splenic tissue to nearly normal appearance. The low-dose APS co-treated group (1.5 mg/kg diet) showed mild deposits of hemosiderin pigment inside splenic tissue with a moderate degree of lymphoid depletion while the high dose APS co-treated group (3 mg/kg diet) showed a better protective effect with only slight hemosiderin deposits and a mild degree of lymphoid depletion (Fig. 13).

### Histopathological scoring

The control group showed well-preserved structures in the gills, muscles, and kidneys, with only a few mild focal changes that were in line with normal biological variability. The total histopathological score for the gills was 0.8 ± 0.6, while for the muscle and kidney it was 1.1 ± 0.7 and 1.5 ± 0.6, respectively. Fish supplemented solely with APS exhibited histological profiles nearly at baseline, thereby confirming the lack of intrinsic toxicity. The 1.5 mg/kg APS-only group had totals of 0.7 ± 0.7 for gills, 0.6 ± 0.5 for muscles, and 1.1 ± 0.6 for kidneys. The 3 mg/kg APS-only group had totals of 0.3 ± 0.4 for gills, 0.1 ± 0.3 for muscles, and 0.7 ± 0.5 for kidneys. On the other hand, exposure to Al_2_O_3_-NPs caused serious histological alterations in examined tissue sections. Gill sections exhibited diffuse epithelial hyperplasia, lamellar fusion, epithelial lifting, and congestion, yielding a cumulative score of 9.8 ± 0.4. Muscle tissue exhibited multifocal degeneration, necrosis, edema, and atrophy, yielding a cumulative score of 9.0 ± 0.7. Kidney sections showed a lot of glomerular atrophy, tubular degeneration, vascular congestion, and epithelial desquamation. The total score was 10.2 ± 0.6. APS supplementation had a clear protective effect that depended on the dose. The low-dose group (APS 1.5 mg/kg diet) showed some improvement, with gill, muscle, and kidney totals going down to 5.2 ± 0.8, 5.3 ± 0.8, and 5.6 ± 0.7, respectively. The high-dose group (APS 3 mg/kgdiet) showed almost complete restoration of cytoarchitecture, with gill, muscle, and kidney totals of 1.5 ± 0.8, 1.5 ± 0.8, and 1.9 ± 0.6, respectively, coming close to the values seen in controls (Table 8).

**Table 8:** Histopathological scores and morphometric area (%).

Organ/parameter	Control	APS 1.5	APS 3	Al_2_O_3_-NPs	APS 1.5 + Al_2_O_3_-NPs	APS 3 + Al_2_O_3_-NPs
Gill score	0.8 ± 0.6	0.7 ± 0.7	0.3 ± 0.4	9.8 ± 0.4	5.2 ± 0.8	1.5 ± 0.8
Muscle score	1.1 ± 0.7	0.6 ± 0.5	0.1 ± 0.3	9.0 ± 0.7	5.3 ± 0.8	1.5 ± 0.8
Kidney score	1.5 ± 0.6	1.1 ± 0.6	0.7 ± 0.5	10.2 ± 0.6	5.6 ± 0.7	1.9 ± 0.6
Liver—hepatocyte vacuolization (%)	1.07 ± 0.25	1.05 ± 0.22	1.04 ± 0.22	41.93 ± 2.16	23.33 ± 1.72	8.86 ± 1.01
Spleen—melanomacrophage center area (%)	2.15 ± 0.21	2.12 ± 0.19	2.08 ± 0.25	18.64 ± 1.38	9.32 ± 1.09	4.71 ± 0.48

### Morphometric analysis

Quantitative morphometric analysis corroborated the histopathological findings. The control group (1.07 ± 0.25%) and APS-only groups (1.5 mg/kg: 1.05 ± 0.22%; 3 mg/kg: 1.04 ± 0.22%) exhibited minimal hepatic vacuolization. Fish exposed to Al_2_O_3_-NPs showed a significantly higher percentage of vacuolated hepatocytes (41.93 ± 2.16%), whereas APS supplementation reduced hepatic vacuolization in a dose-dependent manner (low-dose: 23.33 ± 1.72%; high-dose: 8.86 ± 1.01%). Similarly, splenic melanomacrophage centers (MMCs) were scarce in the control (2.15 ± 0.21%) and APS-only groups (1.5 mg/kg: 2.12 ± 0.19%; 3 mg/kg: 2.08 ± 0.25%), but markedly increased in Al_2_O_3_-NPs–exposed fish (18.64 ± 1.38%). APS supplementation significantly reduced MMCs in a dose-dependent manner (low-dose: 9.32 ± 1.09%; high-dose: 4.71 ± 0.48%) (Table 8).

## Discussion

The use of metal nanoparticles in the aquatic ecosystems has been increased through fish feed, medicines, water treatment processes, packaging, biofilm control, pond and cage sterilization. Subsequently, these metal nanoparticles persist in water and can induce detrimental effects on fish body^55^. Therefore, there is an urgent need to mitigate their damaging effects by using naturally and ecofriendly herbal extracts as APS. Up to date no studies have evaluated the mitigation role of APS against Al_2_O_3_-NPs-induced toxicity in Nile tilapia. Our results highlighted new insights on the mitigative role of APS against Al_2_O_3_-NPs-induced toxicity. The protective effect of APS may be attributed to its flavonoid and phenolic components, which can modulate redox homeostasis and interact with metal ions, thus reduce oxidative stress^56,57^. Additionally, *A. membranaceus* has been reported to restore the redox homeostasis and alleviate cytokine production, contributing to its immunomodulatory properties^58,59^. These mechanisms might be responsible for the observed reduction in fish toxicity caused by Al_2_O_3_-NPs.

The observed reduction in growth performance following Al_2_O_3_-NPs exposure coincides with previous reports by Abd El Megeed et al.^51^ and Yu et al.^60^, who showed that *O. niloticus* exposed to toxic doses of Al_2_O_3_-NPs exhibited significant growth impairment. This decrease may primarily result from oxidative stress induced by Al_2_O_3_-NPs exposure as oxidative imbalance is negatively correlated with growth performance indices^61^. Also, the retardation in the growth performance may be attributed to the impaired water quality and reduced feed utilization efficiency^62^. Under these stress conditions, fish may be forced to expand their energy toward detoxification process rather than somatic growth^63^. On the other side, dietary supplementation with APS significantly improved the growth performance in agreement with previous studies in Nile tilapia, bluegill sunfish, *Larimichthys crocea,* and rice field eels (*Monopterus albus*)^64–68^. Similarly, Farag et al.^33^ reported that the co treatment with APS effectively alleviated sub-lethal thallium toxicity in Nile tilapia. These growth-promoting and protective effects may be attributed to its natural antioxidative and immunomodulatory properties. The growth-promoting effect of APS may be mechanistically linked to improved nutrient assimilation efficiency. APS has been shown to stimulate digestive enzyme activity, promote intestinal villi elongation, and enhance beneficial microbiota colonization, collectively increasing protein synthesis capacity and feed utilization efficiency^35,69–71^. In contrast, Song et al.^72^ reported no significant differences in growth performance in sea cucumber following dietary ASP supplementation. These differences likely reflect species-specific metabolic rates, variation in gut microbiota responsiveness to polysaccharides, and differences in experimental duration and basal diet composition^73^.

In our study, the significant increase in alanine aminotransferase (ALT) activity observed in fish groups exposed to Al_2_O_3_-NPs is consistent with Hadi et al.^74^ who reported increased ALT activity in adult Tilapia zilli following aluminum exposure. Similarly, Abdel-Khalek et al.^10^ and Massoud et al.^75^ reported that exposure to Al_2_O_3_-NPs in *O. niloticus* induced liver damage, as evidenced by the elevated plasma liver enzyme activities. The increase in ALT levels may be attributed to enhanced oxidative stress and subsequent hepatocellular injury, leading to increased membrane permeability and enzyme leakage into circulation. Dietary supplementation with APS significantly ameliorated the liver damage and reduced plasma ALT activity. Similar hepatoprotective pattern have been consistently reported in yellow catfish, crucian carp, and spotted sea bass^44,76,77^. Similarly, Zhou et al.^78^ found that APS alleviated liver injury in rat suffered from induced alcohol liver disease. These hepatoprotective properties of APS are likely mediated through the potent anti-stress effects which preserve cellular integrity and attenuate oxidative damage^65^. However, our results are in contrast with those obtained by Sun et al.^79^ who showed that *Astragalus membranaceus* root extract caused an increase in liver enzymes such as ALT and AST indicating liver damage with no significant effect on liver morphology or hepatocyte integrity in hybrid grouper. Disparities in fish species, the kind or concentration of *Astragalus membranaceus* extract used, as well as changes in experimental design and environmental conditions, could all be responsible for the differences between the studies.

Our results also revealed a significant decrease in some immunological parameters like total proteins (TP, ALB, and GLO) and IgM, in the Al_2_O_3_-NPs-exposed group. This decrease may be due to the liver dysfunction as lower level of total protein is a pointer of hepatic dysfunction^80^. In agreement with our findings, Elkhadrawey et al.^81^ reported reduction in total protein levels along with elevation in ALT and AST levels in rat plasma injected with Al_2_O_3_-NPs. This effect could be explained by the damage in the hepatic tissue, excessive production in ROS, and increase the oxidative stress^82^. On the other side, fish groups supplemented with APS showed significantly increase in the total proteins and IgM level which is in the same trend with previous studies in Largemouth Bass, Coral Trout, and Lined Seahorse^83–85^. This immunostimulatory response may be attributed to synthetic function and support immune defense mechanisms^85^.

Antioxidant defense systems play a vital role in maintaining redox balance and protecting cells from oxidative damage by scavenging excess reactive oxygen species (ROS). Key enzymes, such as superoxide dismutase (SOD) and catalase (CAT), are essential components of this defense system, contributing to cellular stability and protection against ROS^86,87^. In our study, the observed changes in *SOD* and *CAT* gene expression point to the oxidative stress induced by Al_2_O_3_-NPs exposure. Our outcome revealed marked downregulations of *SOD* and *CAT* gene expression in fish groups exposed to Al_2_O_3_-NPs reflecting impaired antioxidant capacity and reduced scavenging efficacy. This reduction is in the same way as Farag et al.^20^ who reported decreased SOD and CAT activities in Nile tilapia exposed to Al_2_O_3_-NPs intoxication. Likewise, Temiz and Kargın^88^ observed a significant decrease (*P* < 0.05) in CAT activity in *O. niloticus* exposed to Al_2_O_3_-NPs. In a different perspective, APS supplemented groups showed significant upregulations in *CAT* and *SOD* gene expression, in agreement with Shao et al.^66^, and Liu et al.^70^ who showed enhanced SOD and CAT activities in *Larimichthys crocea* juvenile following APS dietary supplementation. This may be explained by its potent antioxidant activity, which enhance antioxidant enzyme activities and suppress lipid peroxidation. Dietary APS supplementation has been reported to enhance SOD, CAT, and GSH-PX activity whereas reducing malondialdehyde (MDA) levels, thereby strengthening antioxidant defense mechanisms^64,89^. In contrast to our outcome, Zhao et al.^68^ reported no noticeable effect on the SOD, CAT activities following dietary incorporation of APS. The differences may be attributed to the fish species, fish feed, fish age, and experimental conditions.

The significant up-regulations in *IL-1β* and *TNF-β* observed in our results in fish groups exposed to Al_2_O_3_-NPs indicates oxidative stress and inflammatory status. These cytokines are important in mediating inflammatory process^90^. As mentioned by Sengul and Asmatulu^91^, nanoparticles-induced toxicity can activate inflammatory signaling pathways, resulting in increased expression of *IL-1β* and *TNF-β*. Our data are in agreement with El-Borai et al.^92^; Farag et al.^20^; and Abd El Megeed et al.^51^ who observed increased inflammatory related gene expression following Al_2_O_3_-NPs exposure in rats and *O. niloticus,* respectively. On the contrary, the simultaneous feeding of APS resulted in restoring the expression levels of these genes toward basal levels, consistent with Lin et al.^44^ and Zhao et al.^68^, highlighting the anti-inflammatory and immunomodulatory properties of APS.

The detoxification processes are achieved by the action of metallothionein (MT) which binds and neutralizes the toxic metal ions or ROS^93^. Based on our findings, Al_2_O_3_-NPs exposure resulted in significant upregulation of *MT* gene expression, likely reflecting increased hepatic accumulation of nanoparticle materials and activation of detoxification process^94^. Conversely, APS supplementation significantly downregulated *MT* gene expression, highlighting the strong antioxidant and metal-chelating properties of *Astragalus membranaceus*^27^.

The gills are the first tissues that respond to waterborne pollutants, as they are the main organ that directly faces these pollutants, thereby disturbing osmoregulatory activity and reducing the uptake of oxygen^94^. In the present study, the histopathological changes observed in the gills of fish treated with Al_2_O_3_-NPs were similar to the findings of Benavides et al.^11^, Murali et al.^13^, and Abd El Megeed et al.^51^. These studies reported severe gill damage in *Carassius auratus, Oreochromis mossambicus,* and *O. niloticus*, respectively. These changes include congestion of the central venous sinus, lamellar fusion, epithelial hyperplasia, hypertrophy, and severe inflammatory cell infiltration. These changes were also confirmed using quantitative scores, where the gill lesion score was significantly increased from 0.8 ± 0.6 in the control group to 9.8 ± 0.4 in the Al_2_O_3_-NPs-treated group (*P* < 0.05). These changes greatly increase the distance between the external water pollutants and the blood, thereby hindering the entry of these pollutants into the fish body^96^. However, the APS co-treatment at both dietary levels showed a marked recovery from the aforementioned impacts, as demonstrated by the reduced lesion scores (5.2 ± 0.8 and 1.5 ± 0.8 for APS 1.5 and 3 mg kg^−1^, respectively) and partial recovery of normal gill architecture. This protective effect of APS aligns with the study of Chen et al.^97^, who reported that APS supplementation of *O. niloticus* challenged with *Aeromonas veronii* showed alleviation of detrimental impacts on gill tissue.

In the muscle tissue, the degenerative changes observed in the Al_2_O_3_-NPs-exposed group were comparable to those observed by Murali et al.^13^, where anomalies in muscle fibers, necrosis, and nuclear changes were observed in *O. mossambicus* after exposure to Al-NPs. Quantitative histopathological scoring also revealed significant changes, where the muscle lesion score increased significantly from 1.1 ± 0.7 in the control group to 9.0 ± 0.7 after Al_2_O_3_-NPs exposure (*P* < 0.05). The supplementation of APS significantly reduced the muscle tissue degenerative changes, where the lesion score decreased significantly to 5.3 ± 0.8 and 1.5 ± 0.8 following APS supplementation with 1.5 mg/kg and 3 mg/kg, respectively, restoring muscle striation almost to normal histological structure. To the best of our knowledge, there is no previous study on the effect of APS on skeletal muscle tissue in fish. However, previous studies have confirmed the strong protective effects of APS on skeletal muscle under different stress conditions. Mou et al.^98^ have found that APS can reduce cigarette smoke-induced muscle dysfunction through the anti-inflammatory action of APS on the NF-κB/p53 signaling pathway. In addition, it was reported that APS can improve the antioxidant status and exercise endurance of rats^99^. Furthermore, Lu et al.^100^ have found that APS can inhibit muscle atrophy and apoptosis induced by dexamethasone and H_2_O_2_ through the activation of the Akt/mTOR signaling pathway.

Regarding the liver, the histopathological changes observed in the liver tissue of Al_2_O_3_-NPs-exposed fish are supported by Abdel-Khalek et al.^10^, Benavides et al.^11^, and Murali et al.^12^, who reported severe histopathological changes such as vacuolization, congestion, and necrosis in the liver of fish exposed to nanoparticles. In the present study, vacuolization of hepatocytes showed a dramatic increase from 1.07 ± 0.25% in the control group to 41.93 ± 2.16% in Al_2_O_3_-NPs-exposed fish. APS supplementation showed a remarkable protective role by reducing hepatocyte vacuolization to 23.33 ± 1.72% and 8.86 ± 1.01% at 1.5 and 3 mg kg^−1^ APS, respectively. This is in agreement with the biochemical results, as the ALT levels were higher, and the total protein levels were lower, indicating that the liver tissue integrity was impaired by the nanoparticles. However, the supplementation of APS significantly alleviated the liver lesions, suggesting that the supplementation of APS has a significant hepatoprotective effect. These results are in agreement with the findings of Huang et al.^77^, where the supplementation of APS significantly improved the liver lesions in the high-fat diet-induced liver damage model. Similarly, Liu et al.^101^ showed that APS supplementation effectively alleviated liver damage induced by chemotherapy in mice. These findings suggest that APS has significant antioxidant properties in maintaining liver structure and function.

Concerning the kidney, the histopathological changes observed in Al_2_O_3_-NPs-exposed fish were similar to those observed in *O. mossambicus* exposed to Al_2_O_3_-NPs by Murali et al.^13^, which showed degenerative changes in kidney tissue with melanomacrophage deposits in the interstitial tissue and severe necrosis in hematopoietic tissue. Moreover, Abdel-Khalek et al.^10^ found degenerative changes in kidney tissue in *O. niloticus* exposed to Al_2_O_3_-NPs. In our study, it was found that renal lesion scores were significantly increased in Al_2_O_3_-NPs-exposed fish compared to the control group (1.5 ± 0.6 to 10.2 ± 0.6; *P* < 0.05), indicating severe damage to kidney tissue. However, APS treatment reduced renal lesion scores to 5.6 ± 0.7 and 1.9 ± 0.6 at 1.5 and 3 mg kg^−1^ APS doses, respectively. Moreover, fish supplemented with 3 mg kg^−1^ APS showed significant restoration in kidney tissue cytoarchitecture with normal morphology. Although there are no studies on the effects of *Astragalus membranaceus* extract on fish renal histopathology, studies on mammals have shown that Astragalus components, such as APS and total astragalus saponins, can reduce renal fibrosis and inflammation in rat models and improve histopathology of the kidney^102^. Moreover, Ma et al.^103^ and Zheng et al.^104^ showed that supplementation with APS has protective effects on renal dysfunction in rats. This study supports the hypothesis that APS can protect the kidney from oxidative stress caused by nanoparticles.

In the spleen, the severe tissue structure distortion found in the Al_2_O_3_-NPs-exposed group can be explained by cytotoxicity and genotoxicity caused by excessive ROS production. Oxidative stress can cause DNA, cellular membrane, and protein oxidation^91,105^. Consistent with the above findings, the melanomacrophage center (MMC) area significantly increased after Al_2_O_3_-NPs exposure, from 2.15% ± 0.21% in the control group to 18.64% ± 1.38%. This indicates that immune and detoxification responses were activated after Al_2_O_3_-NPs exposure. The supplementation of APS significantly decreased the MMC area, which reached 9.32% ± 1.09% and 4.71% ± 0.48% after APS treatment at 1.5 mg/kg and 3 mg/kg, respectively, indicating the improvement of tissue structure integrity in the spleen. This is consistent with the findings of Pan et al.^106^, who found that APS supplementation significantly enhanced the integrity of splenic cells of rainbow trout by reducing tissue damage, apoptosis, upregulating immune genes, and inhibiting viral replication in the spleen.

The combination of biochemical, molecular, and histopathological data enables the development of a comprehensive mechanistic framework that explains how APS protects against toxicity caused by AlO_3_-NPs. The current scientific investigation demonstrates that dietary APS significantly plays a protective role in alleviating Al_2_O_3_-NPs-induced toxicity by an antioxidant, anti-inflammatory, and immunomodulatory mechanism. Severe growth inhibition, reduced feed efficiency, and survivability were significantly correlated with Al_2_O_3_-NPs-induced toxicity, which caused marked oxidative stress, immune system disruption, and histopathological damage in multiple target organs. The significant downregulation of antioxidant enzyme genes, including *CAT* and *SOD*, along with the upregulation of *MT*, indicate overwhelming oxidative stress and attempts by the body to cope with metal-induced oxidative damage. At the same time, the significant upregulation of inflammatory markers, including *TNF-β* and *IL-1β*, and the downregulation of serum IgM levels indicate a high level of inflammatory response along with immune system compromise. These significant changes in the molecular mechanism led to severe histopathological damage in the gills, liver, kidney, muscle, spleen, and overall physiological system.

In contrast, APS co-treatment resulted in a marked restoration of redox homeostasis, as supported by the upregulation of *CAT* and *SOD* expression, as well as the downregulation of *MT* gene expression. In addition, APS has demonstrated its efficacy in suppressing inflammatory pathways, as supported by the downregulation of *TNF-β* and *IL-1β* expression. Meanwhile, APS has also demonstrated its efficacy in boosting humoral immunity, as supported by elevated IgM levels and improved splenic histoarchitecture. These molecular and immunological improvements are paralleled by remarkable histopathological improvement in various organs. Therefore, it can be concluded that APS supplementation resulted in improving growth performance, feed conversion efficiency, and survivability.

Notably, the marked biological activity of APS could be mechanistically explained by its high content of phytochemicals, which were characterized by HPLC, showing that it contains flavonoids and phenolics. These bioactive molecules were found to exert effective free radical scavenging, metal chelating, and anti-inflammatory effects, which could be utilized to effectively inhibit oxidative stress and inflammation, especially by affecting NF-κB pathways^51–55^. Additionally, its potential to stimulate immune responses could be useful in restoring immune homeostasis and increasing resistance against diseases. Overall, these multifaceted biological effects of APS could be attributed to its synergistic molecular mechanisms, which could be useful in assessing its potential therapeutic value as a natural feed additive against nanoparticle-induced toxicity and for increasing fish health and productivity.

Taken together, our results gave new visions about the protective and modulatory role of APS against Al_2_O_3_-NPs toxicity in *O. niloticus*. Dietary supplementation with APS high dose, especially at 3 mg/kg, was effective in alleviating the detrimental effects of Al_2_O_3_-NPs toxicity through improving the antioxidant defense mechanisms, restoring the histopathological changes, and downregulating inflammatory and stress-related genes. The strong antioxidant, anti-inflammatory, and cytoprotective activities of APS may contribute to its flavonoid and phenolic content. Collectively, this work highlights the importance of integrating natural immunomodulators like APS into aquaculture practices to reduce the impact of nanoparticle pollution and promote sustainable fish production.

Regardless of these promising results, some limitations should be clarified. First, it should be noted that tissue accumulation of Al_2_O_3_-NPs was not evaluated, which would have helped to better understand dose–response bioaccumulation of Al_2_O_3_-NPs-induced toxicity. Second, it should be noted that this study was conducted under controlled laboratory conditions for a short period of time; therefore, long-term studies should be conducted to validate the efficacy of APS in aquaculture practices. Third, immune challenge test was not conducted; however, this test should be conducted to assess whether APS supplementation would be effective in disease resistance after pathogen challenge.

## Conclusion

Collectively, the current study shows that dietary APS can ameliorate Al_2_O_3_-NPs-induced toxicity in *Oreochromis niloticus* through antioxidant, anti-inflammatory, and immunomodulatory pathways. Al_2_O_3_-NPs exposure was found to impair growth performance, induce hepatic damage, downregulate antioxidant gene expression, trigger inflammatory responses, and cause histopathological alterations in multi-organ systems. However, dietary APS supplementation, especially at 3 mg/kg, was found to alleviate Al_2_O_3_-NPs-induced toxicity through antioxidant pathways, anti-inflammatory pathways, and immunomodulatory pathways.

From an ecological point of view, the current study demonstrates the potential of APS as a sustainable and eco-friendly nutritional intervention strategy to enhance *O. niloticus* health and fitness under nanoparticle-polluted aquatic conditions. With the current increasing incidence of nanoparticle contaminants in aquaculture systems, dietary APS supplementation might be used as an alternative strategy to alleviate nano-toxic stress, ensure fish health, and promote eco-friendly aquaculture production.

## Declarations

## Data Availability

The datasets generated and/or analyzed during the current study are available from the corresponding author on reasonable request.
